# Quantitative assessment of the robustness of next-generation sequencing of antibody variable gene repertoires from immunized mice

**DOI:** 10.1186/s12865-014-0040-5

**Published:** 2014-10-16

**Authors:** Victor Greiff, Ulrike Menzel, Ulrike Haessler, Skylar C Cook, Simon Friedensohn, Tarik A Khan, Mark Pogson, Ina Hellmann, Sai T Reddy

**Affiliations:** Department of Biosystems Science and Engineering, ETH Zürich, 4058 Basel, Switzerland

## Abstract

**Background:**

Next-generation sequencing (NGS) of antibody variable regions has emerged as a powerful tool in systems immunology by providing quantitative molecular information on polyclonal humoral immune responses. Reproducible and robust information on antibody repertoires is valuable for basic and applied immunology studies: thus, it is essential to establish the reliability of antibody NGS data.

**Results:**

We isolated RNA from antibody-secreting cells (ASCs) from either 1 mouse or a pool of 9 immunized mice in order to simulate both normal and high diversity populations. Next, we prepared three technical replicates of antibody libraries by RT-PCR from each diversity scenario, which were sequenced using the Illumina MiSeq platform resulting in >10^6^ 250 bp paired-end reads per replicate. We then assessed the robustness of antibody repertoire data based on clonal identification defined by amino acid sequence of either full-length VDJ region or the complementarity determining region 3 (CDR3). Leveraging modeling approaches adapted from mathematical ecology, we found that in either diversity scenario both CDR3 and VDJ detection nears completeness indicating deep coverage of ASC repertoires. Additionally, we defined reliability thresholds for accurate quantification and ranking of CDR3s and VDJs. Importantly, we show that both factors–(i) replicate sequencing and (ii) sequencing depth–are crucial for robust CDR3 and VDJ detection and ranking.

**Conclusions:**

In summary, we established widely applicable experimental and computational guidelines for robust antibody NGS and analysis, which will help advance systems immunology studies related to the quantitative profiling of antibody responses following infection and vaccination.

**Electronic supplementary material:**

The online version of this article (doi:10.1186/s12865-014-0040-5) contains supplementary material, which is available to authorized users.

## Background

Antibody-secreting cells (ASCs), plasmablasts and plasma cells, play a pivotal role in immunological protection, and thus, are studied intensely in the fields of basic humoral immunity, vaccine development, and monoclonal antibody engineering [1-6]. The ensemble of secreted serum antibodies, IgG representing the predominant isotype [7], constitutes the highly diverse polyclonal antibody repertoire capable of recognizing and specifically binding to many different antigens. Primary antibody heavy chain diversity is achieved by the stochastic rearrangement of three exons (V, D, and J) [8,9]; additional secondary diversification can occur in activated B-cells via somatic hypermutation of the variable (VDJ) region. Antibody-specificity is believed to be dominated by the junctional site of VDJ recombination, also known as the complementarity determining region 3 (CDR3) [10]. The CDR3 has thus served for a long time as a natural identifier of antibody clonality. However, it has recently been suggested that antibody specificity is a result of the close interplay of different parts of the entire VDJ region [11,12]; consequentially, the number of reports relying on the entire VDJ region as clonal identifier is expanding [4,13-16].

An emerging systems immunology method to quantitatively assess the antibody repertoire’s immense diversity is high-throughput immune repertoire analysis, which combines next-generation sequencing (NGS), bioinformatics, and statistical analysis of variable regions [17-19]. In particular, antibody repertoire NGS has become a powerful tool to quantitatively address fundamental questions in immunology related to lymphocyte development and differentiation [20], discovery of clinical diagnostics based on antibody sequence biomarkers [14,21,22], and antibody repertoire diversity [20,23-25]. One of the principal advantages of antibody NGS is the quantitative determination of clonal diversity and distribution, which provides valuable insight into clonal selection and expansion during humoral responses [26]. This assessment of clonal diversity and distribution offers new approaches for vaccine profiling and monoclonal antibody discovery and engineering [1,4,13,27-31].

Due to current technological limitations, the antibody repertoire diversity at any given time can at best only be estimated and not empirically determined in mammals [32,33]. Therefore, the number of sequencing reads to accurately and reproducibly represent diversity information is unclear [34]. Until recently, NGS using the 454 technology led to read counts in the range of 10^4^–10^5^ [4,35], thus most likely undersampling B-cell and ASC numbers (>10^6^) in both mice and humans [36-39]. The advent of the 250 bp paired-end read technology developed by Illumina offers for the first time the possibility to assess antibody repertoire diversity by enabling (i) coverage of the entire VDJ region and (ii) the generation of large numbers of reads (>10^6^) at a more practical cost.

**Figure 1:**
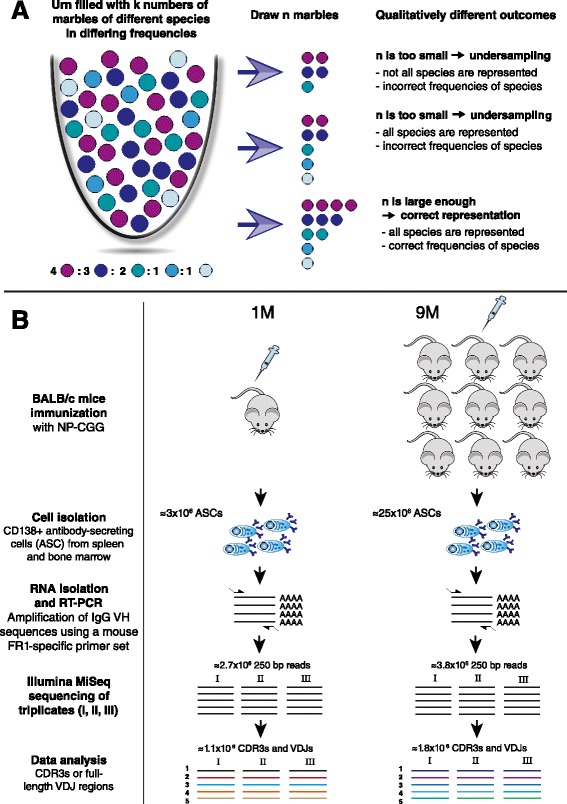
**Motivation and experimental setup. (A)** The problem of undersampling in NGS antibody repertoire sequencing is most easily explained by the marble analogy. Assuming an urn is filled with k numbers of marbles of different species in varying frequencies–urn and marbles represent the original antibody mixture. The problem is clear: if only a sample of size n (n < k) is drawn, then three qualitatively different sampling outcomes can arise: (1) If n is too small, species richness (number of different colors in the urn) is not accurately determined and consequently neither are species frequencies. (2) In the case that n is larger, species richness is accurately represented but species frequencies can be off. (3) Only if n is large enough, both species richness and frequency are accurately reflected in the sample. This study set out to answer, which outcome best describes antibody repertoire NGS data from ASCs of immunized mice. **(B)** To address undersampling concerns, we explored two different scenarios of ASC diversity: 10 female BALB/c mice were immunized with NP-CGG and sacrificed 14 days post-injection. Subsequently, bone marrow plasma cells were isolated as described previously, as were CD138-positive splenocytes [4]. ASCs of 1 mouse (1M) were pooled as were those of 9 mice (9M). RNA was isolated, followed by RT-PCR and Illumina MiSeq sequencing of triplicates (see *Methods*).

Reproducible sequencing of antibody repertoires is of paramount importance for the development of diagnostic approaches [40,41]. In light of recent findings that antibody clonal abundance is correlated to antigen specificity [4,42,43], reliable capture of ranking information is necessary for monoclonal antibody discovery and profiling of antibody responses to vaccines and infections. The concerns of undersampling the antibody repertoire naturally lead to the following questions [17,40]: (i) To what extent are antibody clones within one sample being detected by NGS? (ii) What percentage of detected clones can be reliably and reproducibly identified? (iii) To what extent do reliably detected clones bear accurate frequency information (Figure 1A)?

For the statistical analysis of antibody NGS datasets, we leveraged long-established concepts of ecological population theory, which have only recently been applied to immunoglobulin repertoire diversity [14,23,44-49]. We found that regarding murine IgG-positive ASC repertoire sequencing approximately ≈ 3×10^6^ 250 bp paired-end reads were sufficient to capture their essential diversity information–i.e. number of different clones and their respective clonal frequency–of CDR3s and full-length VDJ regions alike. Triplicate sequencing enabled us to efficiently call reliably detected clones as well as define a threshold for reliable clonal ranking.

This study establishes that NGS of antibody repertoires of immunized mice is a robust technique for exploring questions of fundamental immunological importance such as antibody diversity in response to antigen-challenge. Finally, we offer experimental and computational guidelines for faithful antibody NGS that are independent of model organism, immunization scheme, and target cell population.

## Results

### High quality Illumina triplicate sequencing of antibody-secreting cells from pooled and individual mouse samples

Two scenarios of immunological relevance were assessed for NGS reproducibility. First, we pooled spleen plasmablasts and plasma cells (CD138-enriched) and bone marrow plasma cells (CD45R-depleted and CD138-enriched [4,50]) of one mouse immunized with the model antigen NP-CGG (chicken gamma globulin [CGG] conjugated to 4-hydroxy-3-nitrophenylacetyl) and sacrificed 14 days post-immunization, hereafter called “1M”. The thus created cell pool contained approximately 3×10^6^ viable ASCs. Second, in order to model extreme antibody diversity, we repeated the same cell isolation procedure from nine immunized mice (hereafter called “9M”) resulting in an ASC pool of approximately 2.5×10^7^ viable cells (Figure 1B).

From isolated cells, we recovered total RNA and used RT-PCR to amplify expressed rearranged IgG variable heavy genes. PCR was performed using a well-characterized and utilized primer set based on variable framework region 1 binding forward primers and one IgG constant heavy region 1 reverse primer (covering all IgG isotypes, Additional file 1 [51]). Similarly to previously published methods [52,53], Illumina adapters were added during the PCR step by using a direct addition approach, which adds adapters at the 5′ end of the gene-specific primer set, thus circumventing the need for ligation of adapters following PCR (Additional file 2). For each of the two diversity scenarios (1M/9M), triplicates were prepared, where a triplicate signifies three separately indexed samples prepared from the same starting cDNA pool; thus, variable region PCR amplification was independently performed in each of the triplicates (see *Methods*). All six samples (triplicates of 1M and 9M) were sequenced using the Illumina MiSeq platform with 250 bp paired-end reads (Figure 1B). Sequencing yielded an average of 3.2×10^6^ raw reads (paired-end, 250 bp) for each replicate with mean quality Phred scores ranging from 33 to 36 (Additional file 3).

Sequences were processed (pairing rates of raw 250 bp reads reached an average of ≈ 92%, Additional file 3) and submitted to the open-access ImMunoGeneTics (IMGT)/HighV-Quest platform [54] in order to obtain full-length VDJ region and CDR3 read annotation.

To account for sequencing errors that could artificially inflate diversity, all CDR3 and VDJ singletons (CDR3 and VDJ amino acid sequences that only occurred once) were excluded prior to any data analysis, as were any reads with CDR3s shorter than 4 amino acids. The average number of thus filtered CDR3s and VDJs for 1M/9M were respectively ≈ 1.1×10^6^/≈1.7×10^6^ (unique: ≈14,000/≈30,000) and ≈ 740,000/≈1.0×10^6^ (unique: ≈54,000/≈112,000), which still represented approximately ≈ 95% and ≈ 61% of pre-filtered CDR3 and VDJ sequences respectively (Additional file 3).

### Antibody repertoire sequencing achieves deep coverage of CDR3 clonal diversity

Throughout the entire study, we defined antibody clonality in two ways: (i) based on identical amino acid sequence of variable heavy chain CDR3 (based on IMGT classification); (ii) based on identical amino acid sequence of full-length heavy chain VDJ region. We focused on the analysis of the heavy chain because it contributes predominantly to antibody diversity and specificity [10].

Frequency distributions of CDR3s and VDJs differed markedly from one another. For each of the diversity scenarios (1M/9M), frequency distributions of VDJs were less polarized than CDR3s (Additional file 4) since for any sample the number of unique VDJs was much higher than the number of unique CDR3s (Additional file 3). Due to an increased CDR3 and VDJ diversity in 9M replicates, cumulative frequency curves saturated faster for 1M than for 9M; this was especially evident for CDR3s (Additional file 5).

**Figure 2:**
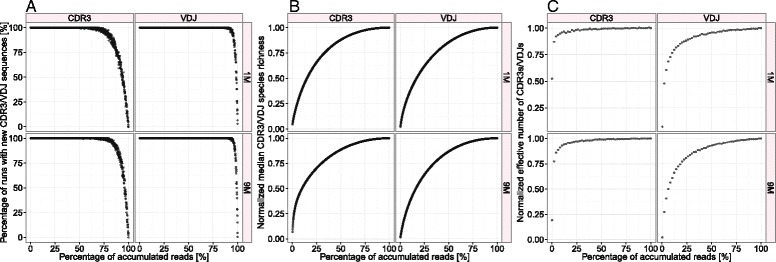
**Diversity information was captured for all CDR3 and VDJ clones with an abundance of two or more regardless of ASC diversity (1M/9M).** To address undersampling, we used a sequential sampling scheme (bootstrapping) in which CDR3 and VDJ datasets were divided into 1,000 read subsets, which were successively added to virtual samples until they had the same size as the original samples. We performed 200 simulation runs for one sample of each diversity scenario (1M/9M) having the highest ratio of different CDR3 clones to total numbers of CDR3s. Graphs show **(A)** the percentage of simulation runs with newly seen CDR3/VDJ clones in a given subset; **(B)** the normalized median CDR3/VDJ species richness; **(C)** normalized effective number of CDR3/VDJ species, where “normalized” signifies the division by the respective maximal value.

Since comparison across replicates depends on their deep coverage, we first addressed the issue of undersampling. To do so, we relied on simulations that used a sequential sampling scheme (bootstrapping) for both CDR3 and VDJ clonal distributions. Simulations were performed for one replicate dataset from the 1M and 9M triplicates. The replicate that was chosen was based on having the unfavorably highest ratio of different CDR3s to total CDR3s. We performed a bootstrapping approach with 1,000 equally sized sampling steps, where in each step a random set of CDR3/VDJ sequences (from the replicate NGS dataset) was added to virtual samples. Sampling steps continued until virtual samples had accumulated the same number of sequences as the starting replicate (Figure 2).

On the basis of these simulations, we sought to plot species accumulation curves, which were determined by calculating the proportion of simulation runs that added new CDR3/VDJ sequences to the virtual samples (Figure 2A). An undersampling issue would be present in samples if new clones were still added at 100% of accumulated sequences [34,45]. However, we found that for both CDR3 and VDJ and both diversity scenarios (1M/9M) the probability of obtaining a new clone nears zero for read accumulation rates ranging between 75% and 100% (Figure 2A). We therefore concluded that repertoire NGS of murine antibody producing cells achieves high coverage of clonal diversity and is not substantially influenced by undersampling.

Complementing species accumulation curves, we also plotted the median species richness of the simulation runs (Figure 2B). Species richness signifies the number of different CDR3/VDJ sequences. Species richness curves of both CDR3s and VDJs leveled off toward 75% of accumulated reads indicating that 75% of the sequences would have been enough to accurately represent a replicate’s species richness.

Lastly, to study how frequency-dependent diversity depends on read accumulation, we assessed the change of the effective number of species as a function of accumulated reads. The effective number of species (ENS) is the exponential of the Shannon entropy, which is a widely used measure of repertoire diversity [44,55]. If all clones are equally abundant, then the ENS is high, tending towards the species richness. Conversely, if one clone dominates the repertoire, then the ENS is low, tending towards 1 [46,55]. In addition to including species richness, the ENS also takes CDR3/VDJ frequencies into account. For CDR3s, nearly the entire ENS information was captured at only 25% of accumulated sequences, whereas for VDJ it required nearly 75% of accumulated sequences for complete ENS coverage (Figure 2C). Curves of 1M leveled off slightly faster than 9M curves.

To summarize, we found that independently of clonal definition, diversity scenario, and statistical method, diversity information was captured at or below 100% read accumulation. In particular, the number of reads needed to exhaustively cover antibody repertoire diversity was considerably reduced if CDR3/VDJ frequency information was taken into consideration.

### Replicate sequencing enables reliable detection of antibody clones

**Figure 3:**
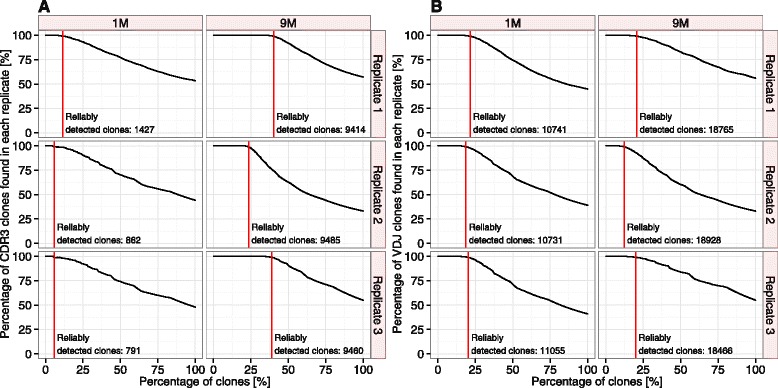
**Establishing a cut-off for reliable clonal detection.** For each replicate (**A**: CDR3, **B**: VDJ), starting with the highest frequency clones, clones were tested for their simultaneous presence in the respective two other replicates. For each clone, presence or absence in the other two replicates were recorded. We regarded all those clones as “reliably detected” that belonged to the highest frequency set of clones with a ratio of presence to absence ≥99%. The percentage of clones passing the detection threshold is indicated by vertical red bars. Absolute numbers of reliably detected clones for CDR3-1M were (1427, 862, 791), and for CDR3-9M were (9414, 9485, 9460). For VDJ, 1M and 9M absolute numbers were (10741, 10731, 11055) and (18765, 18928, 18466), respectively. For a graphical overview of the method of reliable detection, please refer to Menzel *et al.* [53].

Leveraging the deep repertoire coverage, we proceeded by establishing a cutoff, which ensures that 99% of clones in a given replicate would be found in the two other replicates (Figure 3). Resulting from this reliable detection cutoff, for 1M triplicates, the top 1427, 862 and 791 CDR3s and for 9M triplicates, the top 9414, 9485 and 9460 CDR3s were found to be reliably detected (Figure 3, Additional file 6). Accordingly, the numbers of reliably detected VDJs for 1M were 10741, 10731 and 11055 and for 9M 18765, 18928 and 18466 (Figure 3, Additional files 3 and 6). The reliably detected CDR3/VDJ clones for each replicate were used in all analyses shown hereafter. Thus, replicate sequencing provided a powerful method to establish reliability of clonal detection.

### High reproducibility of antibody repertoire sequencing

**Figure 4:**
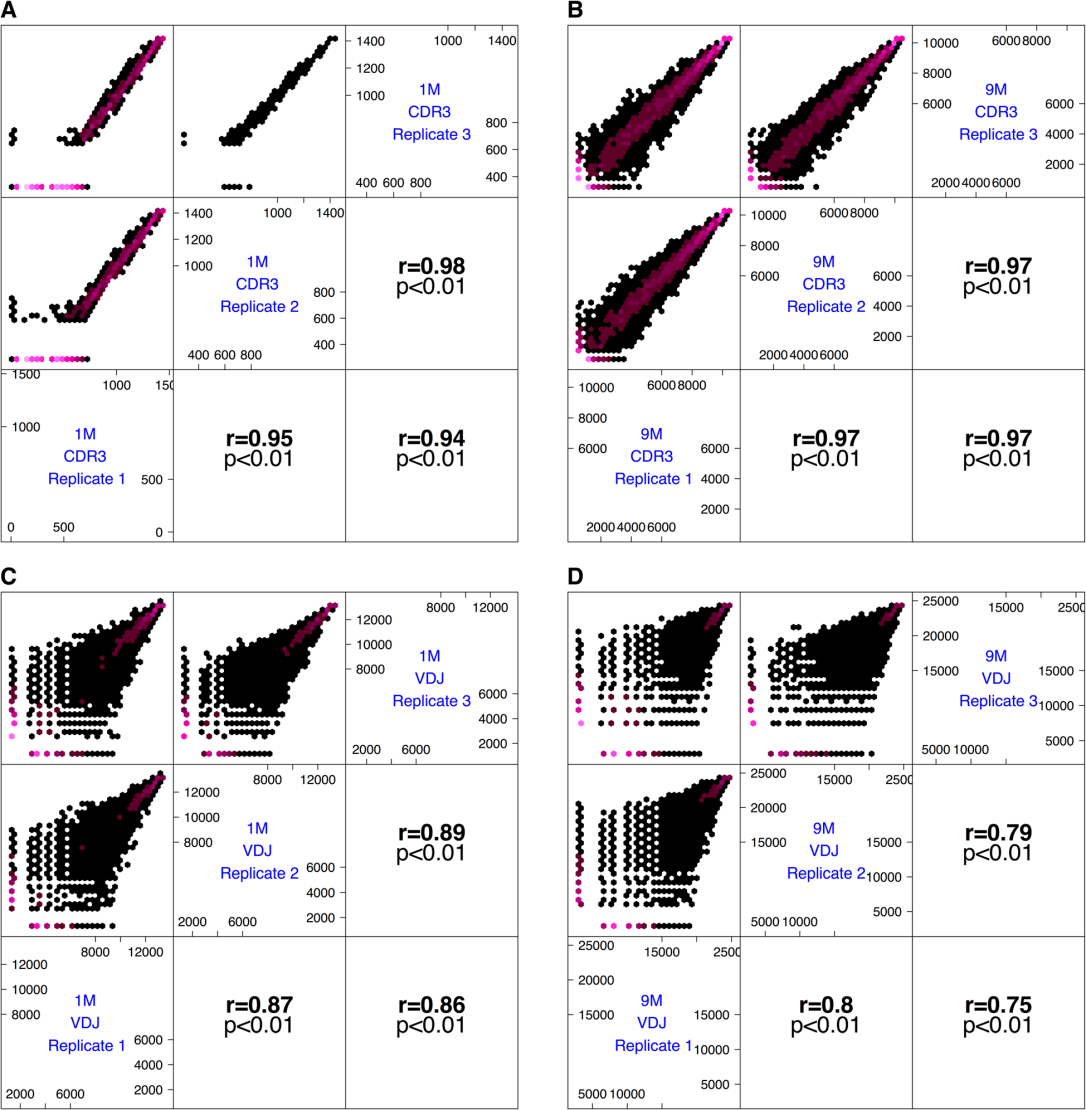
**Correlation of ranks of CDR3 and VDJ sequences between replicates is high (CDR3: r ≥ 0.94, VDJ: r ≥ 0.75) demonstrating reproducibility of antibody repertoire sequencing.** The ranks of CDR3 (**A**: 1M, **B**: 9M) and VDJ (**C**: 1M, **D**: 9M) sequences were determined by assigning the highest rank to the CDR3 or VDJ sequence with the highest abundance, the second highest rank to the CDR3 or VDJ sequence with the second highest abundance and so forth. Association of ranks was determined using Pearson correlation (r). To circumvent overplotting, correlation plots are displayed using hexagons–purple indicates where data points accumulate. Only reliably detected CDR3 and VDJ sequences (Figure 3) were considered for the analysis shown.

Due to the high importance of reliable clonal ranking for antibody discovery and vaccination studies [4], clonal CDR3/VDJ frequencies were converted to ranks to check for reproducibility across triplicates. The highest rank was attributed to the CDR3/VDJ identical amino acid sequence with the highest frequency, the second highest rank to the CDR3/VDJ with the second highest frequency and so forth. Rank-converted triplicates were Pearson correlated in a pairwise fashion, which led to CDR3 correlation coefficients of r > 0.93 (range: r = 0.94–0.98, p < 0.001) and VDJ correlation coefficients of r > 0.74 (range: r = 0.75–0.89, p < 0.001) for both diversity scenarios (1M/9M) (Figure 4). Accordingly, correlations between triplicates based on CDR3 and VDJ frequencies had very high coefficients (r > 0.98 for both 1M and 9M, Additional file 7). Q-Q plots also showed that in addition to ranking and frequency, clonal distributions were highly reproducible, thereby emphasizing the high fidelity of Illumina MiSeq-generated antibody NGS data (Additional files 8 and 9).

### Deep coverage of antibody repertoires is crucial to obtain reliable ranking of antibody clones

**Figure 5:**
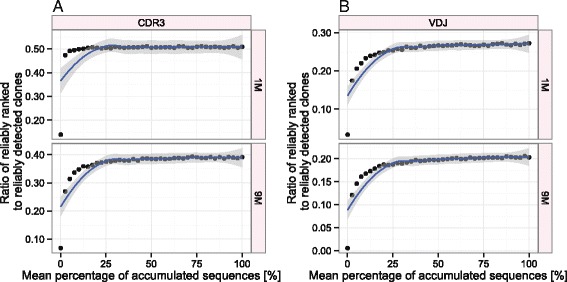
**Reliable ranking of (A) CDR3 and (B) VDJ sequences depends on deep sequence coverage of samples.** Plots show the ratio of reliably ranked clones–expressed as the median number of clones having a rank coefficient of variation (CV) lower than 0.05–to the number of reliably detected clones determined in Figure 3 as a function of the proportion of accumulated reads. A LOESS curve was fitted to the calculated ratios (displayed as points) in order to extrapolate their overall distribution with a 0.95 confidence interval (gray-shaded area). As species richness increases, the maximum value of the ratio decreases. Across diversity scenarios (1M/9M) and clonal definitions (CDR3/VDJ) the respective maximum is reached at 25% of accumulated reads. Simulations were performed analogously to those shown in Figure 2 using a sequential sampling scheme (bootstrapping). The CV was calculated based on ranks of CDR3/VDJ clones across triplicates. Absolute numbers of reliably ranked clones were: CDR3: 730 (1M), 4160 (9M); VDJ: 3708 (1M), 5169 (9M).

In light of previous research demonstrating a correlation between clonal ranking and specificity [4], we assessed the reliability of ranking as a function of sequence coverage. We defined reliability of ranking information as the coefficient of variation (CV = SD/mean) of CDR3/VDJ ranks across triplicates. Hence, in contrast to pairwise correlation coefficients, reliability of ranking information took advantage of all three replicates for a given diversity scenario. We deemed the rank of a CDR3/VDJ reliable if its CV was below 0.05, which we used as a reliability cutoff (Additional file 10). Similarly to Figure 2, we used a sequential sampling scheme to assess the dependence of sequencing depth on rank reliability by calculating the ratio of the number of reliably ranked CDR3s/VDJs to the number of reliably detected CDR3s/VDJs as a function of accumulated reads. Simulations showed that the maximum ratio of reliably ranked sequences was achieved at ≈ 25% of accumulated reads for both definitions of clonality (CDR3/VDJ) and both diversity spectra (1M/9M, Figure 5). Ratios of reliably ranked clones are positively proportional to the number of different clones found per number of reads reaching a maximum of ≈ 50% for CDR3-1M and a minimum of ≈ 21% for VDJ-9M (Figure 5, Additional file 10). Thus, with increasing CDR3/VDJ diversity, the percentage of reproducibly detected clones that can be reliably ranked decreases.

In summary, triplicate sequencing does not only enable reliable calling but also ranking of antibody clones: notably, we show that both factors–(i) replicate sequencing and (ii) sequencing depth–are important for a high reliability of clonal detection and ranking.

## Discussion

NGS of antibody variable region repertoires has begun to make a major impact on the emerging field of systems immunology by providing a quantitative assessment of humoral immune responses. In this study, we assessed in great detail the robustness and reproducibility of NGS antibody amplicon data in a common experimental setting. To render our results relevant to a wide range of research groups, we used the common experimental setting of spleen and bone marrow ASC from immunized mice. ASCs are of great immunological interest [5,7,56-61] as they represent the effector cell population of the humoral immune system producing the vast majority of circulating IgG antibodies, which are responsible for immediate and long-term protection against pathogens [56,57]. Experimentally, we adapted previously established methods for ASC isolation and generation of antibody libraries for NGS [4,13,52,53]. As a result of recent improvements in read length of the Illumina MiSeq platform, we were able to sequence full-length VDJ regions. These improvements in sequencing technology were critical for the execution of this study and are increasingly being adopted for repertoire sequencing [14,62,63]. For data analysis, we used statistical approaches that have been first developed in ecological sciences [64,65] but could be readily transferred to NGS, as questions regarding species discovery are fundamental problems encountered in both disciplines. These approaches have been recently applied to B- and T-cell repertoire analyses by other groups [14,44,45,66].

Specifically, our strategy consisted of using mouse ASC populations after primary immunization from both normal diversity (1M) and a high diversity (9M) scenario to quantify clonal diversity and distributions of CDR3 and VDJ amino acid sequences. We isolated ≈ 3×10^6^ ASCs in the 1M sample and ≈ 2.5×10^7^ ASCs in the 9M sample by magnetic bead-enrichment (Figure 1). Since only ≈ 30–50% of these cells are estimated to be IgG-positive [67], the sequencing pool had an estimated size of 1×10^6^ and 7×10^6^ for 1M and 9M, respectively. The high diversity 9M scenario led to a less polarized distribution of CDR3s and VDJs compared to 1M (Additional files 3, 4, and 5). Importantly, the overlap between 1M and 9M datasets was marginal suggesting minimal or no contamination across samples (Additional file 6). Biologically interpreted, the small overlap between 1M and 9M datasets (Additional file 6) suggests that the antibody repertoires of the inbred mice immunized with a medium-complexity antigen (NP-CGG) contained few shared clones. The small overlap of expressed antibody repertoires between individuals existing in a rather controlled environment has been previously shown by others [23], including genetically identical immunized mice of the same cohort [4]. Therefore, the 9M sample mirrored in fact a highly diverse antibody repertoire.

Our analysis revealed the following major points: (i) With an average of ≈ 3×10^6^ 250 bp reads per replicate, antibody repertoire NGS provided *deep sequence coverage* over a wide diversity range (1M/9M) with respect to multiple definitions of clonality (CDR3 or full-length VDJ amino acid sequence); (ii) leveraging the deep repertoire coverage and the sequenced triplicates allowed for the establishment of *unambiguous reliability cutoffs for clonal detection*; (iii) down-stream analysis with reliably detected clones demonstrated the *reproducibility of both clonal detection and clonal frequency distributions*; (iv) the reliability of CDR3/VDJ ranking (ranking being one of the chief indicators of antigen-specificity [4,43]) was not achieved to the same extent as clonal detection. Furthermore, the percentage of reproducibly detectable CDR3s that can at the same time be reliably ranked decreased as clonal diversity increased (Additional files 3 and 9) supporting the intuitive result that more diverse samples require greater sequencing depth for sufficient coverage.

We obtained reliable ranking information for approximately 21–50% (1M/9M) of the reliably detected CDR3 and VDJ species richness (Figures 3 and 5). These accurately ranked sequences may be very valuable for monitoring the clonal selection and expansion that takes place following vaccination or primary infection. To further increase the resolution of clonal ranking, a dramatically increased number of reads would be necessary. Assuming both a constant number of reliably detected clones and a linear relationship between the number of reliably ranked clones and sequencing depth, two (51%, CDR3-1M, Figure 5A) to five (19%, VDJ-1M, Figure 5B) times more reads would be necessary to reliably rank all reliably detected clones. Indeed, Toung and colleagues report that regarding RNA-sequence analyses of human B-cells, a five times higher read coverage is necessary to accurately represent frequencies of reliably detected transcripts [20,68,69]. However, the above estimations should be regarded as lower bounds, as with increasing sequencing depth the number of reliably detected clones increases (until reaching a maximum) and the relation between sequencing depth and ranking information is of non-linear nature. In fact, the impact of undersampling on ranking accuracy may in part explain why frequency-based discovery of antigen-specific monoclonal antibodies was successful when applied to polarized bone marrow plasma cells from immunized mice [4] but was unsuccessful when applied to the less polarized total splenocyte population from immunized rabbits [31].

Recently, Vollmers and colleagues, performed NGS of human B-cell repertoires from vaccinated patients, in which they used RNA template-barcoding to decrease errors introduced during PCR and Illumina sequencing [13]. Their approach dramatically reduced false positive species richness and especially eliminated to a large part singletons–which we found comprised 73–85% of unique sequences depending on the clonal definition–suggesting that our singletons are mostly due to sequencing error. In agreement with procedures adopted by other groups [13,70], singletons were thus rightly eliminated from any data analysis conducted in this report without dramatically reducing the overall size of the datasets (Additional file 9). In addition, we showed that removal of singletons does not substantially impact ENS (increasing CDR3 species richness from 25% read accumulation onward in Figure 2B does not entail an increase in ENS in Figure 2C). However, it should be noted that in cases of very low coverage or read depth the removal of singletons may artificially decrease diversity. Apart from technological limitations, the biological significance of very rare sequences is unclear and remains to be elucidated [71].

While first and foremost our findings are valid for murine ASCs collected from a specific mouse strain immunized once with a single antigen, implications regarding the dependence of the completeness of species richness and frequency information on ASC diversity can be readily transferred to many other antibody repertoire sequencing studies. From the above conclusions, a generally valid framework in form of practical guidelines for the analysis and reliable information extraction from antibody NGS datasets emerges. (i) Immunized mice can be sequenced with deep coverage yielding–in any diversity scenario–reliable detection and ranking of a minimum of at least 20% of reliably detected clones (both CDR3 and VDJ, 1M/9M). This represents a higher number of candidate (reliably detected and ranked) clones than previously published [4,43]. Importantly, the concept of reliable detection scales with the available sequencing depth; higher sequencing depth will result in higher numbers of reliably detected clones. Indeed, Figures 3 and 5 provide a direct assessment of the relation of sequencing depth and the extent to which reliable sequencing and ranking can be established. More generally, by providing NGS data for two definitions of antibody clonality in two different diversity scenarios, we provided benchmark and orientation values thus guiding NGS studies performed with other cell populations and/or other species, accordingly. (ii) If one desires to perform robust antibody repertoire NGS, it is advantageous (and now affordable) to perform replicates per condition (e.g., “healthy”, “immunized”) in order to set thresholds of reliability for both CDR3 detection and ranking. It is reassuring that replicate sequencing is sufficient to achieve high reliability in antibody NGS in the presence of both PCR error and Illumina sequencing error. Indeed, it has been shown recently that the number of replicate samples significantly improves detection power–even more so than sequencing depth [72,73]. (iii) Statistical methods borrowed from theoretical ecology are especially useful if facing samples with unknown diversity, which is to this date still the case for all antibody NGS studies but especially pronounced in repertoire analyses of non-FACS-sorted human PBMC B-cell populations [13,43]. Importantly, diversity measures incorporating frequency information saturate faster for a given number of reads (Figure 2C), allowing for meaningful diversity comparisons across samples even in the case of limited read numbers. Nevertheless, we believe that the issue of biological undersampling in human samples still poses a challenge deserving further attention, as due to practical and ethical reasons it is typically only possible to obtain small fractions of human B-cells (usually from PBMCs), which thus may not accurately represent the overall humoral immune status [17,40].

Recently, mRNA/cDNA barcoding using unique molecule identifiers (UMIs) [74-77] has been used for error correction in immune repertoire NGS studies [13]. However, the extent to which the use of UMIs reproducibly decreases technological noise and increases the recovery of biological information is as of yet unknown [78]. Studies involving UMIs are dependent on the deep coverage of UMI diversity to ensure meaningful consensus-read formation [13,63]; UMI diversity is introduced via degenerate nucleotide regions within reverse transcription (and first-strand synthesis) primers, which itself may be a source of bias [52]. Therefore, UMI studies face potential PCR bias and undersampling problems both on the clonal and UMI-tagging level. Our above formulated framework does not assume any prior knowledge on sample preparation and is thus independent of experimental (e.g. RNA/DNA barcoding) and bioinformatical pre-processing steps. Therefore, the framework’s guidelines may be readily applied to studies incorporating UMI data correction and to further increase quantitation and reproducibility. Applying statistico-ecological methods to UMI-tagged datasets would allow crucial insight into the relation of sequencing depth and the extent of error correction. As of yet, subjective sequence cutoffs have been used to reduce the influence of technological noise on cross-sample comparisons [13]. In contrast, our concept of reliable detection and ranking relies on the explicit exploitation of replicate sequencing: it yields a list of reliably detected clones, which collectively define an unbiased range of detection reliability (e.g. CDR3 sequences 1–1427, 1M, Figure 3A). Therefore, our framework offers the possibility to be applied to all immunoglobulin (antibody or TCR) NGS studies, including those relying on UMIs as an additional step for error correction in order to determine upper and lower bounds of reliable detection and ranking. The definition of ranges of reliable detection and ranking may be valuable for drug and vaccine development [72].

## Conclusions

The ability to robustly detect and rank antibody sequences is highly valuable for future investigations in systems immunology; specifically those in which accurate clonal diversity and distributions are critical such as in infection or vaccination studies [13,28,79-81]. Now that antibody diversity information is being captured at a deep level, studies setting out to define antibody signatures of health and disease [21,82] are justified as are studies taking advantage of diversity measures to compare antibody diversity across individuals [28].

## Methods

### Mouse immunizations

All animal experiments were performed under the guidelines and protocols approved by the Basel-Stadt cantonal veterinary office (Basel-Stadt Kantonales Veterinäramt Tierversuchsbewilligung #2582). Female BALB/c mice (n = 10, Charles Rivers Laboratories) 6–8 weeks old were housed under specific pathogen-free conditions and were maintained on a normal chow diet.

Purified chicken gamma globulin (CGG) conjugated to 4-hydroxy-3-nitrophenylacetyl (NP, NP-CGG, BioCat) was resuspended in sterile-filtered phosphate buffered saline (PBS) at 1.0 mg/mL. On the day of primary immunization, 50 μl of NP-CGG solution was mixed with 100 μL of Alum adjuvant (1 mg/mL, Invivogen) and 50 μL of sterile PBS and stored on ice. The NP-CGG Alum mixture was injected with a 26-gauge needle subcutaneously into the backpad. Mice were sacrificed on day 14 after primary immunization and blood, spleen, and bone marrow (femora and tibiae) were collected.

### Cell isolation

Single cell suspensions from total spleen and bone marrow were obtained as described previously [4]. Briefly, tibiae, femora, and spleens were collected into RPMI supplemented with 10% fetal bovine serum (FBS, Sigma, medium 1). Bone marrow cells were flushed out using a 26-gauge syringe (Braun) and spleens were disintegrated using syringe and forceps. Cells were filtered through a 70-μm cell strainer (BD) and subsequently centrifuged at 1,500 rpm for 10 min at 4°C. Red blood cells were lysed for 3 min in red blood cell lysis buffer (eBioscience). The cell suspension was then washed once with 10 mL medium 1. Finally, cells were resuspended in 1 mL PBS supplemented with 0.5% bovine serum albumin (BSA) and 2 mM ethylenediaminetetraacetic acid (EDTA, buffer 1). Bone marrow cells were depleted of CD45R-positive cells before plasma cell isolation by incubation with anti-CD45R-biotin antibody (eBioscience, 13-0452) for 15 min on ice. After washing the cell suspension twice in BSA-free PBS supplemented with 2 mM EDTA (buffer 2), 30 μL of washed streptavidin-coupled Dynabeads (M-280, Invitrogen) were added and incubated for 20 min on ice. Following magnetic isolation, the negative fraction was used in the subsequent steps.

For plasma cell enrichment, spleen and CD45R-depleted bone marrow cell fractions were incubated with anti-CD138-biotin antibody (BD, 553713) for 15 min on ice. Following two washing steps in buffer 1, 30 μL of Dynabeads were added and incubated for 20 min on ice as before. Bead-bound cells were manually counted on a cell counter (Neubauer), buffer removed by magnetic separation, and beads plus cells were lysed in 1 mL of TRIzol reagent (Invitrogen) and stored at -80°C until further usage. Following cell isolation, isolated spleen and bone marrow ASCs were combined from nine mice (9M, ≈2.5×10^7^ total ASCs) and one mouse (1M, ≈3×10^6^ total ASCs) samples.

### Preparation of IgG genes ready-to-use for next-generation sequencing

Total RNA was extracted using the PureLink RNA Mini Kit (Life Technologies), according to the manufacturer’s protocol. RNA concentration was measured on a Nanodrop 2000c Spectrophotometer and RNA integrity and concentration were further evaluated on a 2100 Bioanalyzer (Agilent Technologies). Isolated ASC RNA from the single mouse and the 9 mice was homogeneously pooled, aliquoted and frozen together at -80°C. Next, cDNA of total mRNA was prepared with half of the total 9M RNA and total 1M RNA using Maxima Reverse Transcriptase (Fermentas) and Oligo(dT) primers (Thermo Scientific) and following the manufacturer’s protocol. For each individual reaction, 500 ng RNA was used, cDNA from multiple reactions were pooled and stored at -80°C.

PCR amplification of the variable heavy IgG genes was performed with a set of 19 forward primers binding in the framework region 1 of the VDJ region as previously described [4,51,53] (Additional file 1) and an IgG-specific reverse primer binding in the constant heavy region 1 (5′ CARKGGATRRRCHGATGGGG 3′). The Illumina TruSeq universal adapter sequence constituted the 5′ portion of the forward primers, while the IgG-reverse primer contained the Illumina index adapter sequence, thereby directly adding Illumina adapter sequences to PCR products (Additional file 2). As cell populations (1M and 9M) were sequenced in triplicates, each PCR sample was prepared with its own unique index primer. As previously described [4], each 50 μL PCR reaction consisted of 0.2 μM of forward primer mix and reverse primer, 5 μL of Thermopol reaction buffer (NEB), 200 μM dNTPs, 2 μL of unpurified cDNA, 0.25 μL Taq DNA polymerase (NEB) filled up with double-distilled water. For each separately indexed sample 10 PCR reactions were run in parallel as follows: 95°C for 3 min; 4 cycles (95°C for 30 sec, 50°C for 30 sec, 68°C for 1 min); 4 cycles (95°C for 30 sec, 55°C for 30 sec, 68°C for 1 min); 20 cycles (95°C for 30 sec, 63°C for 30 sec, 68°C for 1 min); 68°C for 7 min; 4°C storage. PCR clean-up was performed in order to reduce the volume and products were run on a 1% agarose gel for purification. Bands of ≈ 550 bp were gel-excised (Additional file 11), purified and libraries were submitted for a final quality control step on a Bioanalyzer 2100 (Agilent, Additional file 12) prior to sequencing.

### Sequencing methods

Read libraries were obtained by 250 bp paired-end sequencing on the Illumina MiSeq platform and can be accessed from the European Nucleotide Archive (http://www.ebi.ac.uk/ena, ENA Study Accession: ERP003950).

MiSeq forward and reverse reads were paired using PANDAseq [83] with default parameters. Successfully paired sequences were sent to IMGT/HighV-Quest [54] for annotation of CDR3 and full-length VDJ regions. For downstream analyses, only those sequences were kept in which (i) the CDR3 and VDJ region could be detected by IMGT and (ii) of which CDR3s were of minimal length of 4 amino acids. Exclusively, CDR3 and VDJ regions with a minimal abundance of 2 read counts were used for downstream analyses unless mentioned otherwise (Additional file 3). CDR3 and VDJ abundance were calculated based on occurrence of exact amino acid sequences.

### Statistics

Association between variables was tested by Pearson correlation unless mentioned otherwise. P-values below 0.05 were regarded as significant.

### Simulations

Bootstrapping was performed using a sequential sampling scheme in which CDR3/VDJ clones were sequentially added to virtual samples (the number of virtual samples being equivalent to the number of simulations performed) to then determine curves of species accumulation, species richness, and effective number of species.

### Diversity measure calculations

Species richness was calculated as the number of different (unique) CDR3/VDJ clones in a dataset. The effective number of species $ENS = exp\ \left(-{\displaystyle {\sum}_{i=1}^n{p}_i \log }\ {p}_i\right)$ was calculated as the exponential of the Shannon entropy of a given frequency distribution as described previously [55], where *p*_*i*_ is the frequency of the *i*th CDR3/VDJ and *n* is the total number of unique CDR3s/VDJs. The ENS ranges from 1, in a sample with only one clone (or a highly dominating clone), to *n*, the total number of unique clones. For entropy calculations, the natural logarithm was used. Normalized measures (Figure 2) were calculated by dividing by the maximum respective value, i.e. the respective value at 100% sequence accumulation.

As a measure of polarization of the ASC repertoire, we chose the Berger-Parker index [84]. It is calculated as the ratio of N_max_/N, where N_max_ is the abundance of the top CDR3/VDJ sequence and *N* the sum of abundances of all CDR3/VDJ in a replicate.

### Software

Starting from IMGT output obtained, data analyses were performed using the R statistical programming environment [85]. Non-base R packages used for analyses were: ggplot2 [86], VennDiagram [87], ShortRead [88], and hexbin [89].

## Additional files

Additional file 1:
**Primer list for the amplification of full-length IgG variable regions.** 19 partly degenerate forward primers specific for framework region 1 of the variable heavy chain were used together with a reverse primer specific for all IgG subclasses. TruSeq universal and index adapter sequences are 5′ of gene-specific regions, allowing for simultaneous variable heavy gene amplification and preparation for Illumina NGS. Illumina diversity regions (5′ of gene-specific regions) were needed by the Illumina software for reliable cluster calling. Additional file 2:
**Primer design for IgG heavy chain amplification allowing simultaneously direct addition of Illumina sequencing adapters.** Forward primers were adapted from Krebber and colleagues [51]. **(A)** The forward primer mix, consisting of 19 (partially) degenerate primers, binds in the framework region 1 of the VDJ region, while the unique reverse primer binds specifically in the IgG constant heavy region 1. All primers contain a sequence of 4 random nucleotides (termed diversity region), which was necessary for cluster identification on the Illumina chip. All forward primers contained the Illumina universal adapter and the reverse primer contained the reverse complement of a given index adapter, which enabled multiplexed sequencing. **(B)** Primer design and binding to the variable (V) region (framework region 1) and constant **(C)** region (constant region 1) cDNA template. Additional file 3:
**Read statistics.** Quality Phred scores, returned 250 bp paired-end reads prior to PANDAseq pairing and IMGT annotation and detected CDR3s/VDJs prior to application of various cutoffs (≥2 and reliability cutoff established in Figure 3). For each cutoff, the total number of CDR3s/VDJs (“All”) as well as respective “unique” CDR3s/VDJs (species richness) are reported. Average numbers are reported as are the percentages of CDR3s/VDJs that passed cutoffs compared to the total number of CDR3s/VDJs. Percentages in brackets indicate (i) the ratio of PANDAseq-paired reads out of the returned 250 bp reads (column: “Returned reads”), (ii) or the ratio of CDR3s/VDJs out of the PANDAseq-paired reads (columns: “All CDR3s/VDJs”). Replicate 2 (1M) and 3 (9M) have been used in simulations shown in Figure 2. Additional file 4:
**CDR3 repertoires from 1M were more polarized than those from 9M and CDR3 repertoires were consistently more polarized than VDJ repertoires (1M, 9M).** The Berger-Parker index was used to measure the polarization of repertoires [84] and is determined as the ratio *N*_max_/*N* , where *N*_max_ is the abundance of the top CDR3 or VDJ clone, and *N* is the sum of abundances of all CDR3 or VDJ sequences in a replicate. The index was calculated both for frequency distributions containing either all CDR3 or VDJ clones with an abundance of 2 or higher and those CDR3 or VDJ clones passing the reliability cutoff established in Figure 3. Additional file 5:
**Frequency and cumulative frequency plots of reliably detected CDR3 (A,B) and VDJ sequences (C,D) by replicate and diversity scenario (1M, 9M) show an exponential distribution of antibody repertoires.** Relatively few different CDR3 or VDJ sequences constitute a large part of sequencing reads. Reliable detection of CDR3 and VDJ sequences was established in Figure 3. Additional file 6:
**CDR3 and VDJ sequence overlap among triplicate sample.** Venn diagrams were compiled based on reliably detected CDR3s **(A, B, C)** and VDJs **(D, E, F)** for each scenario or respective mean abundance distributions (1M/9M). Reliably detected CDR3 and VDJ sequences were determined as detailed in Figure 3. Low overlap between mean distributions (1M/9M) indicates minimal or no cross-contamination. Mean distributions were determined by averaging the abundance of each CDR3 or VDJ across replicates of a given diversity scenario (1M/9M). Additional file 7:
**Triplicate correlation of CDR3 and VDJ frequencies is high.** CDR3 **(A, B)** and VDJ **(C, D)** frequencies were Pearson correlated (r) among triplicates (CDR3: r ≥ 0.9926, VDJ: r ≥ 0.9871) indicating the reproducibility of antibody repertoire sequencing. Only reliably detected CDR3 and VDJ sequences (Figure 3) were considered for the analysis shown. Additional file 8:
**Quantile-Quantile (Q-Q) plots of triplicates show that CDR3 frequency distributions (Additional file** **5** **) are reproducible across diversity scenarios.** Q-Q plots (1M: A, B, C; 9M: D, E, F) represent a graphical approach for comparing two frequency distributions by plotting their quantiles against each other. If the two distributions being compared are similar, the quantiles will lie on the indicated line. Only reliably detected CDR3 sequences (Figure 3) were considered for the analysis shown. Additional file 9:
**Quantile-Quantile (Q-Q) plots of triplicates show that VDJ frequency distributions (Additional file** **5** **) are reproducible across diversity scenarios.** Only reliably detected VDJ sequences (Figure 3) were considered for the analysis shown (1M: A, B, C; 9M: D, E, F). Additional file 10:
**Determination of reliability of CDR3 and VDJ ranking.** **(A, B)** Taking advantage of triplicate sequencing, coefficients of variation (CV = SD/mean) based on ranks were determined for all reliably detected CDR3 or VDJ clones (as determined in Figure 3) and plotted in function of highest to lowest mean CDR3/VDJ frequency. CDR3/VDJ having a CV lower than 0.05 were regarded as reliably ranked and are shown in red. Absolute numbers of reliably ranked clones are: CDR3, 730/4,160 (1M/9M), VDJ, 3,708/5,169 (1M/9M). Additional file 11:
**Triplicate IgG amplicon libraries on 1% agarose gel for (A) 1M and (B) 9M diversity scenarios.** Libraries were prepared each with cDNA equivalent to 500 ng of total pooled RNA of ASCs (see *Methods*). 570-bp sized amplicons were gel-extracted and purified. Legend: M – 100 bp DNA ladder. Additional file 12:
**Bioanalyzer electropherograms of (A) one 1M and (B) one 9M replicate after gel extraction.** Single, clear peaks were detected for all triplicates of both diversity scenarios (other replicates not shown), resulting on average in 450/1,200 pg NGS-ready library for 1M/9M, respectively.
