# Supplementary material for: Quantitative assessment of the robustness of next-generation sequencing of antibody variable gene repertoires from immunized mice
Source: BMC Immunol. 2014 Oct 16;15:40. doi: 10.1186/s12865-014-0040-5 (PMC4233042; doi:10.1186/s12865-014-0040-5)
Supplement: Additional file 1: — Primer list for the amplification of full-length IgG variable regions. 19 partly degenerate forward primers specific for framework region 1 of the variable heavy chain were used together with a reverse primer specific for all IgG subclasses. TruSeq universal and index adapter sequences are 5′ of gene-specific regions, allowing for simultaneous variable heavy gene amplification and preparation for Illumina NGS. Illumina diversity regions (5′ of gene-specific regions) were needed by the Illumina software for reliable cluster calling. [file 12865_2014_40_MOESM1_ESM.docx]

| **IgH Universal Forward** | **Universal Adapter sequence + Diversity region + VH 5' specific Region** |
| --- | --- |
|  |  |
| IgH-UAd-fw1 | Universal Adapter - NNNN GAKGTRMAGCTTCAGGAGTC |
| IgH-UAd-fw2 | Universal Adapter - NNNN GAGGTBCAGCTBCAGCAGTC |
| IgH-UAd-fw3 | Universal Adapter - NNNN CAGGTGCAGCTGAAGSASTC |
| IgH-UAd-fw4 | Universal Adapter - NNNN GAGGTCCARCTGCAACARTC |
| IgH-UAd-fw5 | Universal Adapter - NNNN CAGGTYCAGCTBCAGCARTC |
| IgH-UAd-fw6 | Universal Adapter - NNNN CAGGTYCARCTGCAGCAGTC |
| IgH-UAd-fw7 | Universal Adapter - NNNN CAGGTCCACGTGAAGCAGTC |
| IgH-UAd-fw8 | Universal Adapter - NNNN GAGGTGAASSTGGTGGAATC |
| IgH-UAd-fw9 | Universal Adapter - NNNN GAVGTGAWGYTGGTGGAGTC |
| IgH-UAd-fw10 | Universal Adapter - NNNN GAGGTGCAGSKGGTGGAGTC |
| IgH-UAd-fw11 | Universal Adapter - NNNN GAKGTGCAMCTGGTGGAGTC |
| IgH-UAd-fw12 | Universal Adapter - NNNN GAGGTGAAGCTGATGGARTC |
| IgH-UAd-fw13 | Universal Adapter - NNNN GAGGTGCARCTTGTTGAGTC |
| IgH-UAd-fw14 | Universal Adapter - NNNN GARGTRAAGCTTCTCGAGTC |
| IgH-UAd-fw15 | Universal Adapter - NNNN GAAGTGAARSTTGAGGAGTC |
| IgH-UAd-fw16 | Universal Adapter - NNNN CAGGTTACTCTRAAAGWGTSTG |
| IgH-UAd-fw17 | Universal Adapter - NNNN CAGGTCCAACTVCAGCARCC |
| IgH-UAd-fw18 | Universal Adapter - NNNN GATGTGAACTTGGAAGTGTC |
| IgH-UAd-fw19 | Universal Adapter - NNNN GAGGTGAAGGTCATCGAGTC |
|  |  |
| **IgG Index Reverse** | **Index X Adapter sequence (RC) + Diversity Region + IgG constant specific region (RC)** |
|  |  |
| IgGall-IdxX-Rv | Adapter Index X - NNNN CARKGGATRRRCHGATGGGG |
