# Supplementary figures and images for: Quantitative assessment of the robustness of next-generation sequencing of antibody variable gene repertoires from immunized mice
Source: BMC Immunol. 2014 Oct 16;15:40. doi: 10.1186/s12865-014-0040-5 (PMC4233042; doi:10.1186/s12865-014-0040-5)

A

## Direct Addition

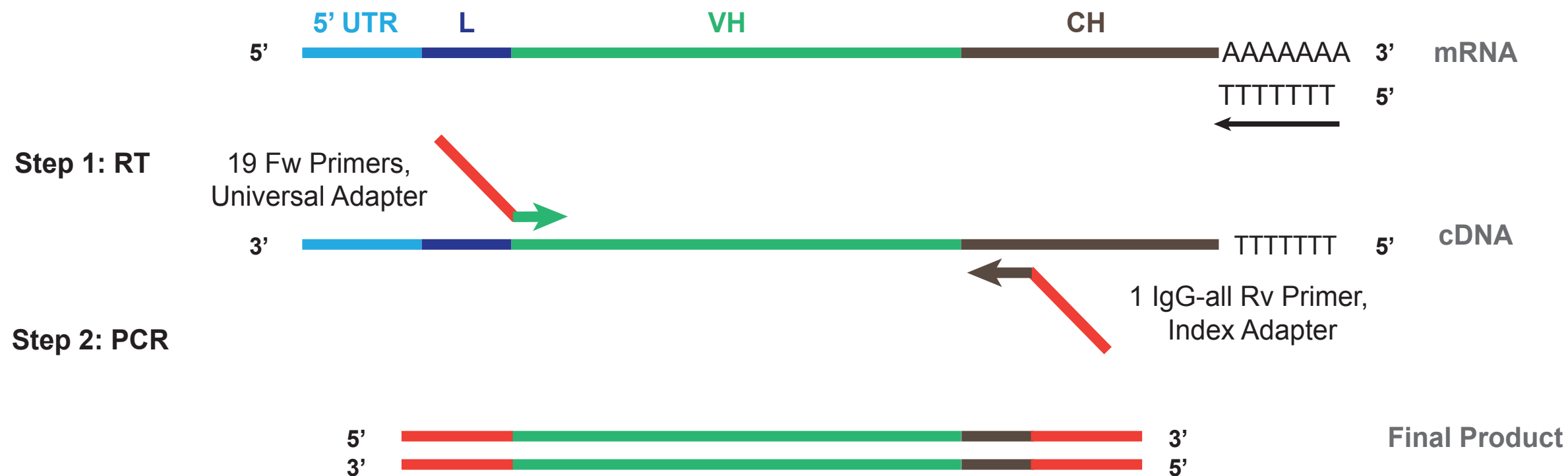

B

## 1 out of 19 Fw Primers

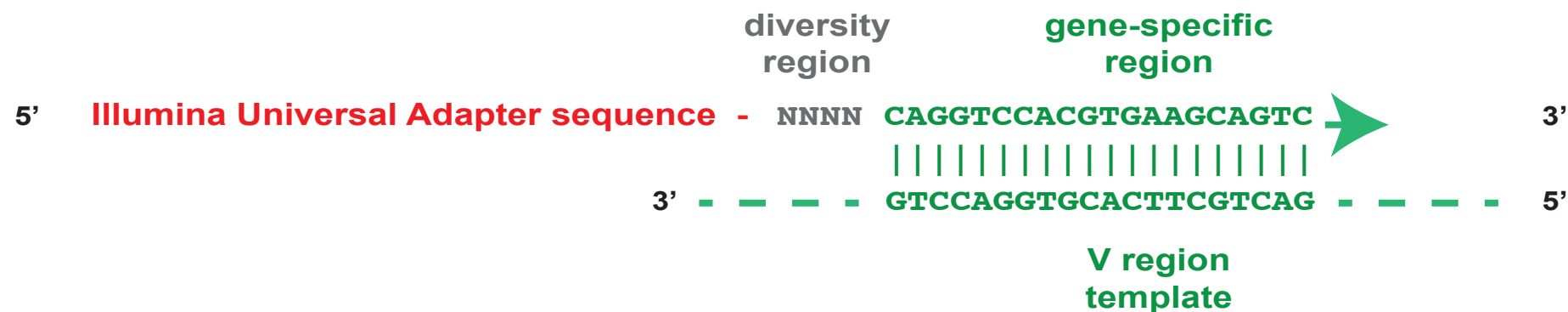

## IgG-Rv Primer

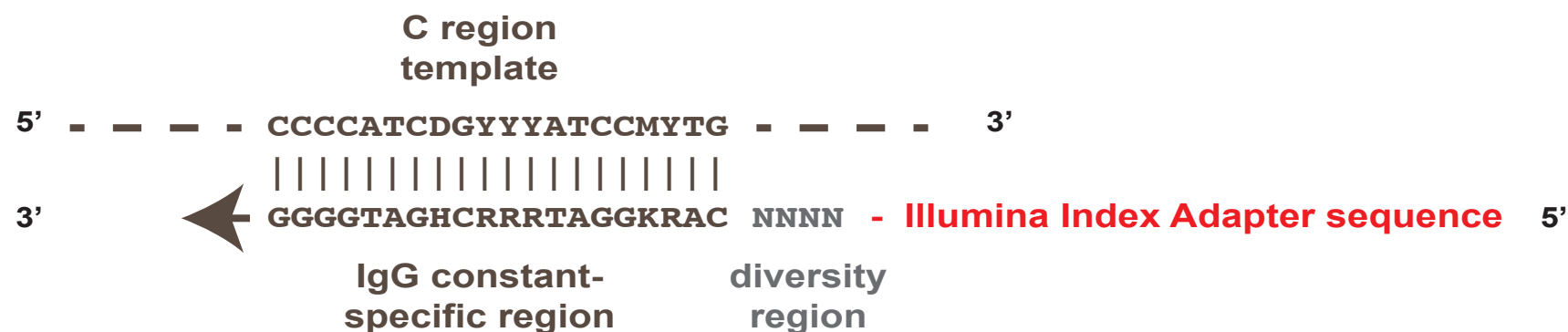

Supplement: Additional file 2: — Primer design for IgG heavy chain amplification allowing simultaneously direct addition of Illumina sequencing adapters. Forward primers were adapted from Krebber and colleagues [51]. (A) The forward primer mix, consisting of 19 (partially) degenerate primers, binds in the framework region 1 of the VDJ region, while the unique reverse primer binds specifically in the IgG constant heavy region 1. All primers contain a sequence of 4 random nucleotides (termed diversity region), which was necessary for cluster identification on the Illumina chip. All forward primers contained the Illumina universal adapter and the reverse primer contained the reverse complement of a given index adapter, which enabled multiplexed sequencing. (B) Primer design and binding to the variable (V) region (framework region 1) and constant (C) region (constant region 1) cDNA template. [file 12865_2014_40_MOESM2_ESM.pdf]

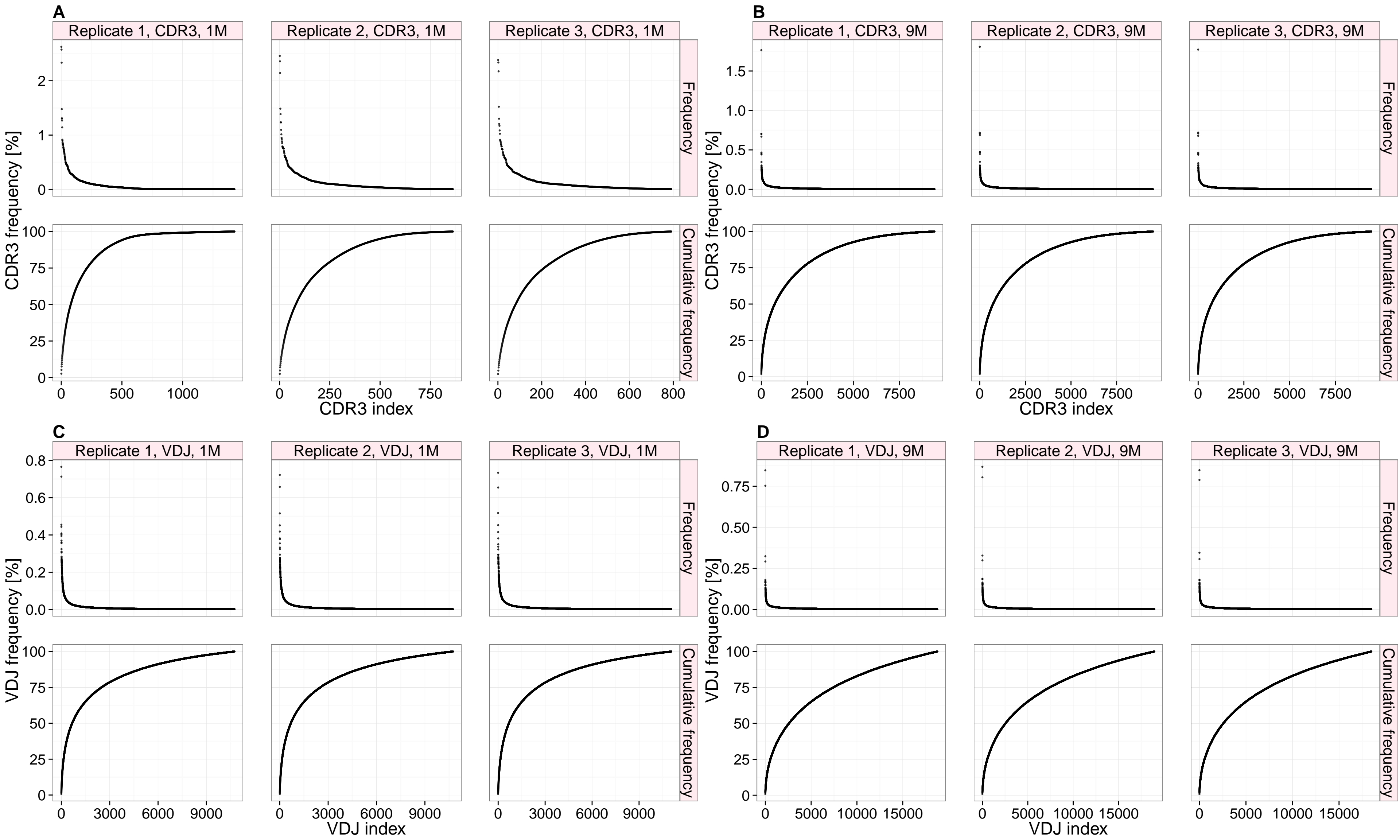

Supplement: Additional file 5: — Frequency and cumulative frequency plots of reliably detected CDR3 (A,B) and VDJ sequences (C,D) by replicate and diversity scenario (1M, 9M) show an exponential distribution of antibody repertoires. Relatively few different CDR3 or VDJ sequences constitute a large part of sequencing reads. Reliable detection of CDR3 and VDJ sequences was established in Figure 3. [file 12865_2014_40_MOESM5_ESM.pdf]

**A**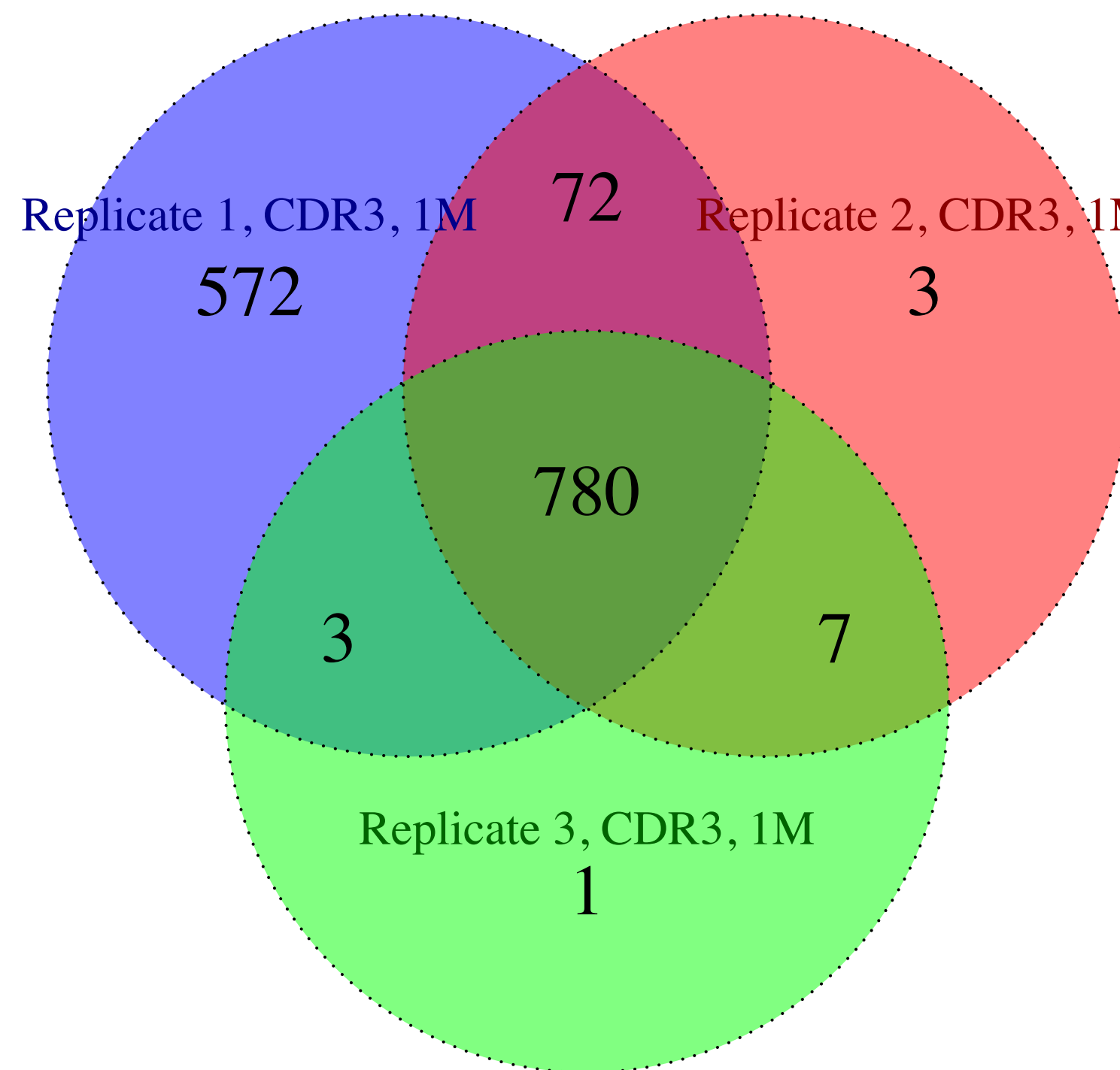**B**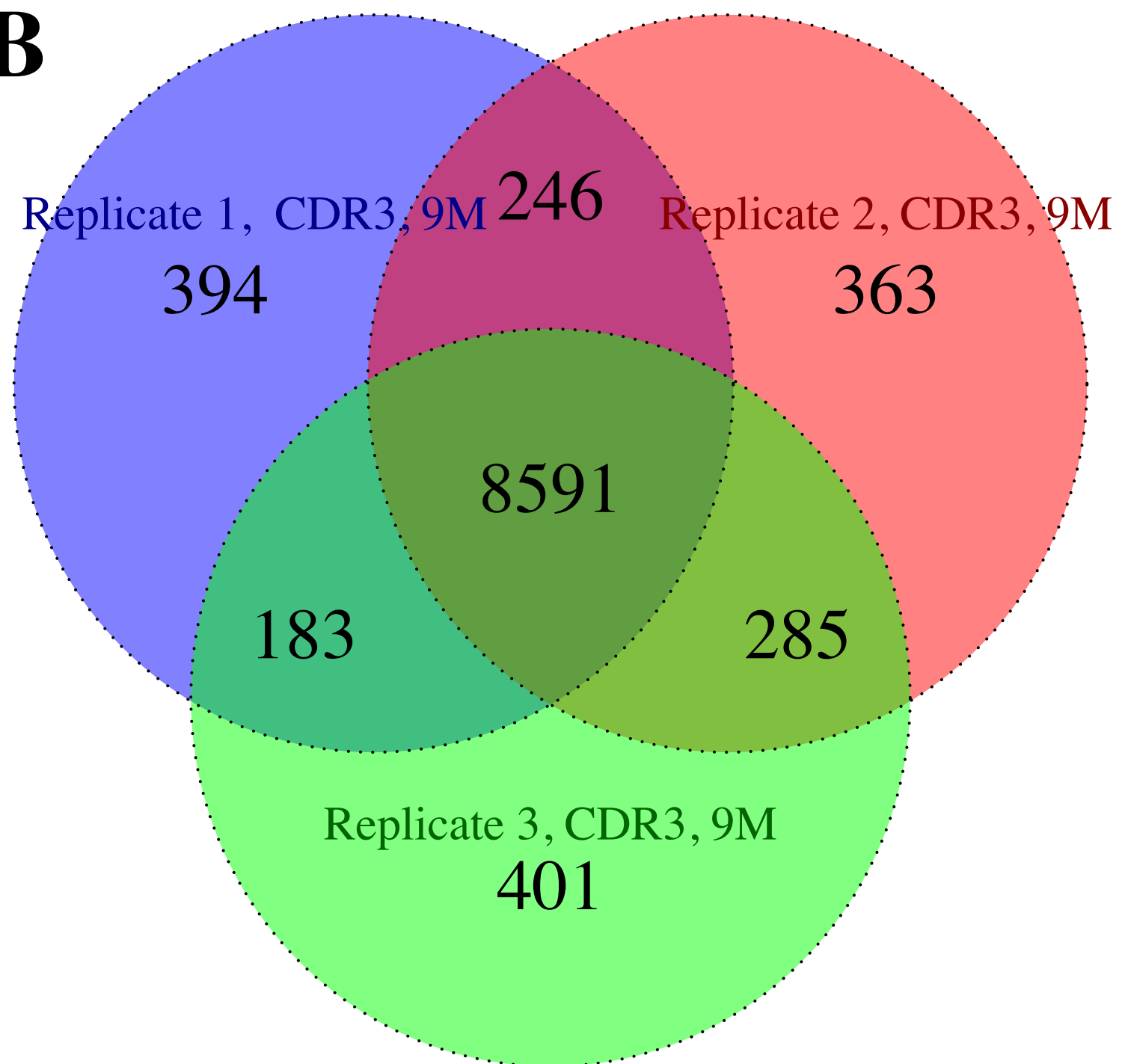**C**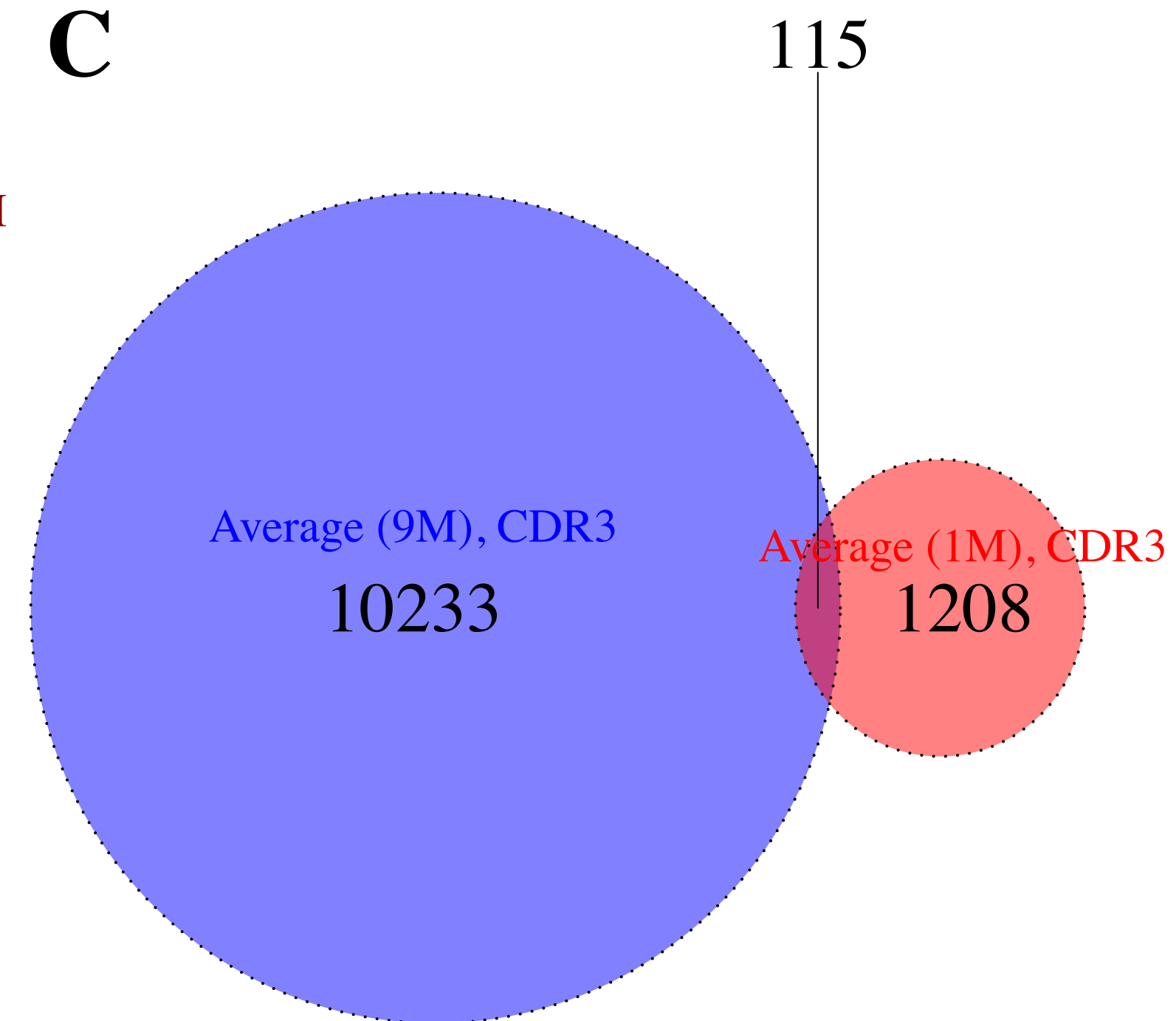**D**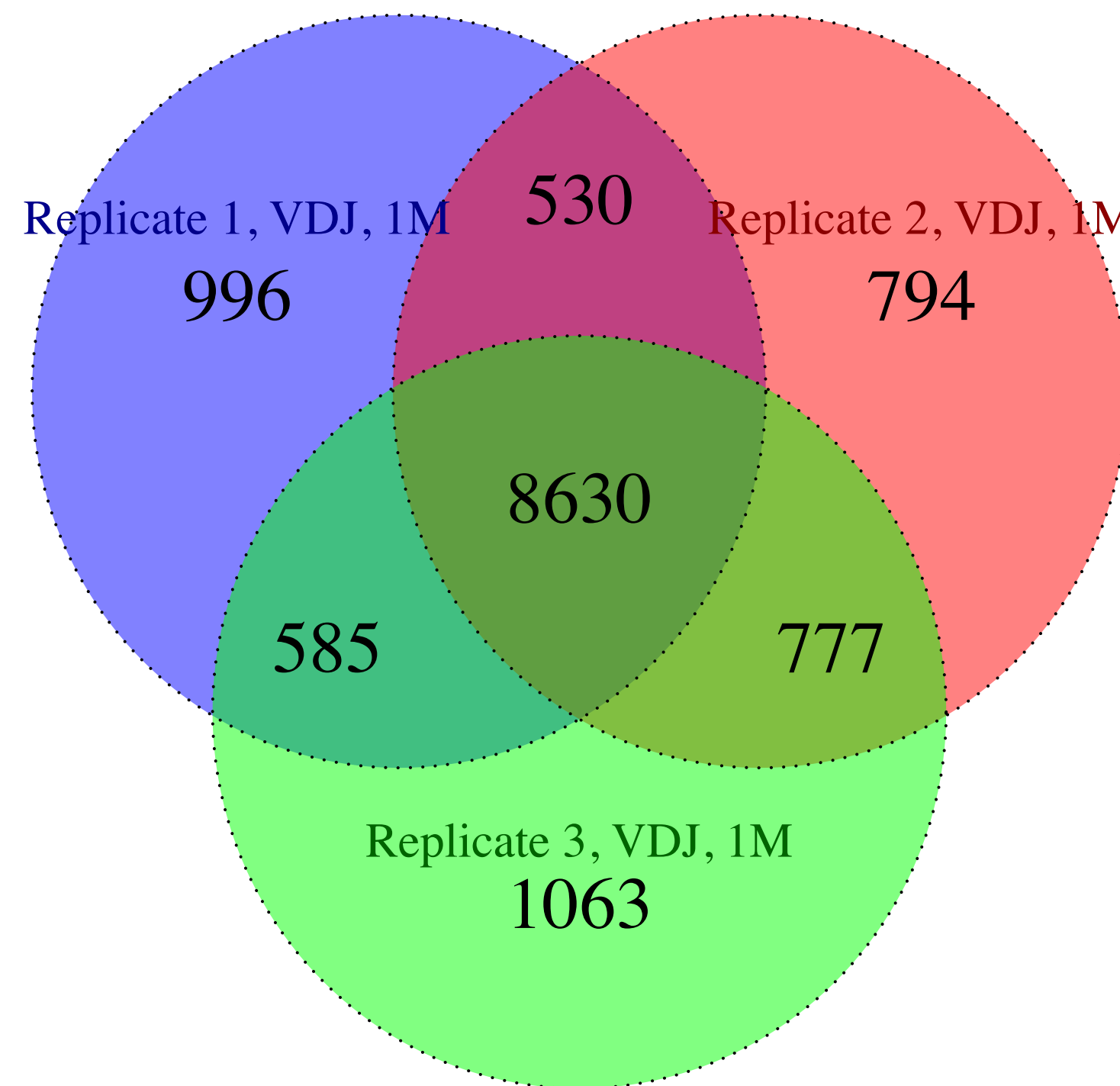**E**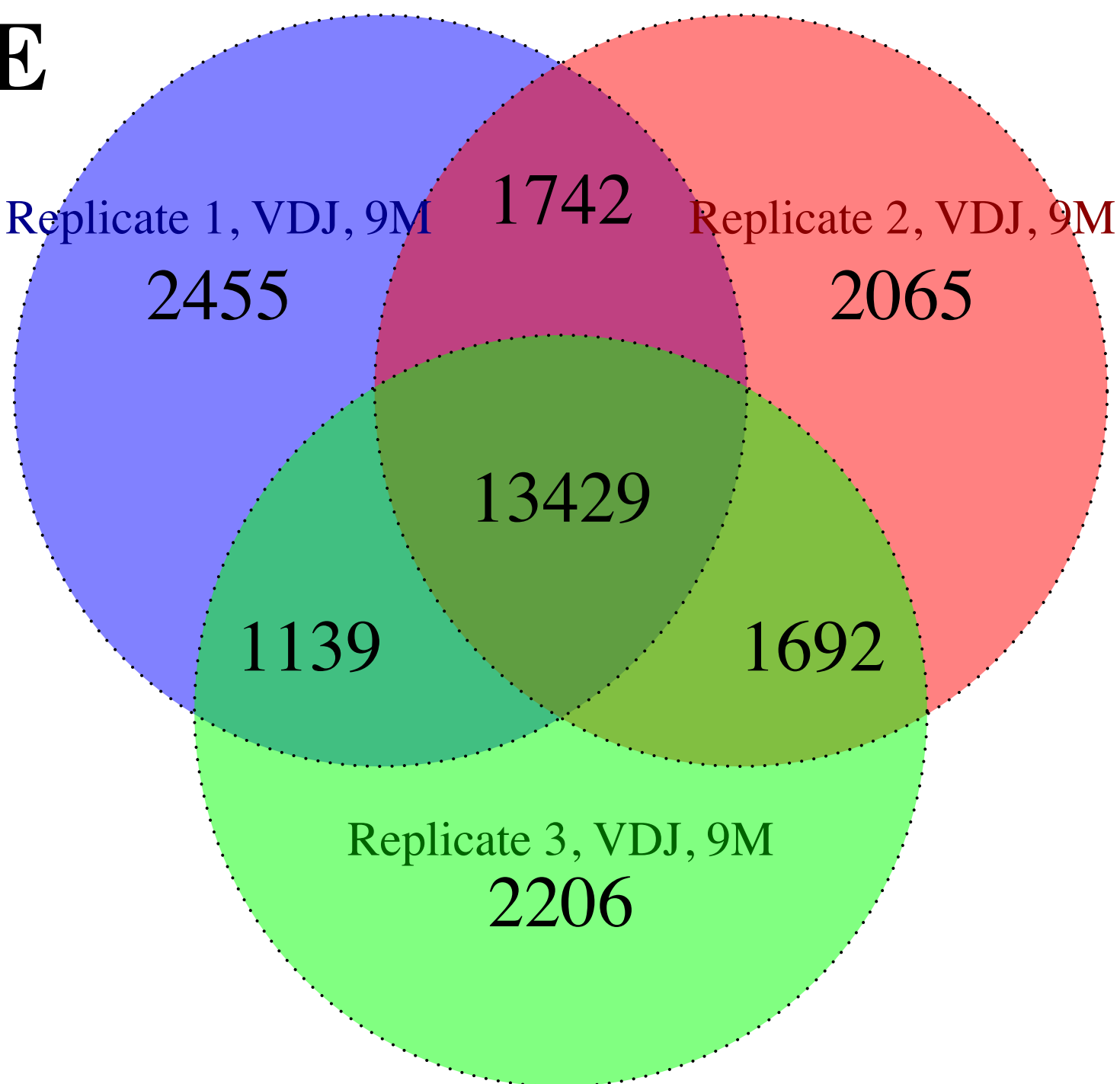**F**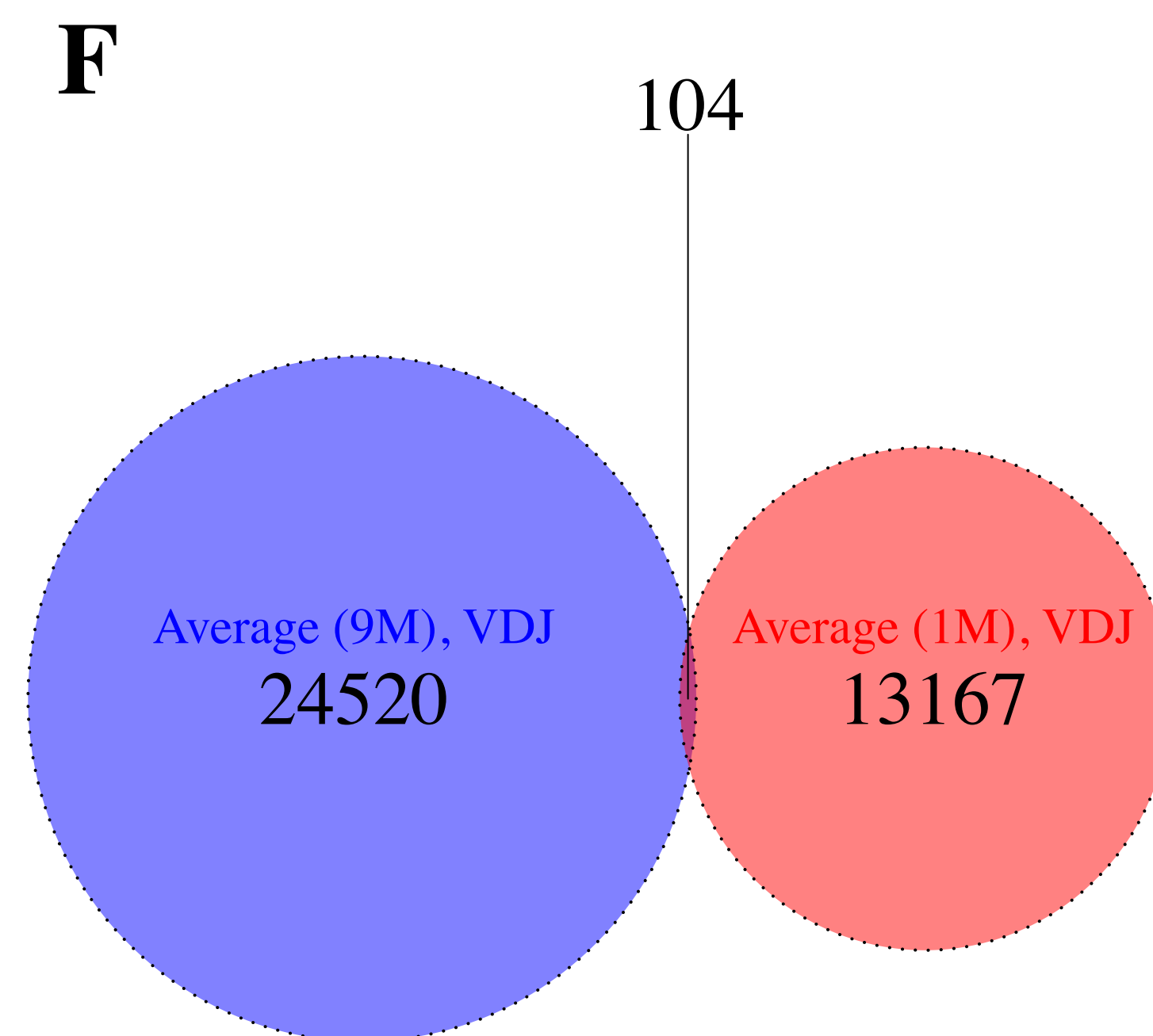

Supplement: Additional file 6: — CDR3 and VDJ sequence overlap among triplicate sample. Venn diagrams were compiled based on reliably detected CDR3s (A, B, C) and VDJs (D, E, F) for each scenario or respective mean abundance distributions (1M/9M). Reliably detected CDR3 and VDJ sequences were determined as detailed in Figure 3. Low overlap between mean distributions (1M/9M) indicates minimal or no cross-contamination. Mean distributions were determined by averaging the abundance of each CDR3 or VDJ across replicates of a given diversity scenario (1M/9M). [file 12865_2014_40_MOESM6_ESM.pdf]

A

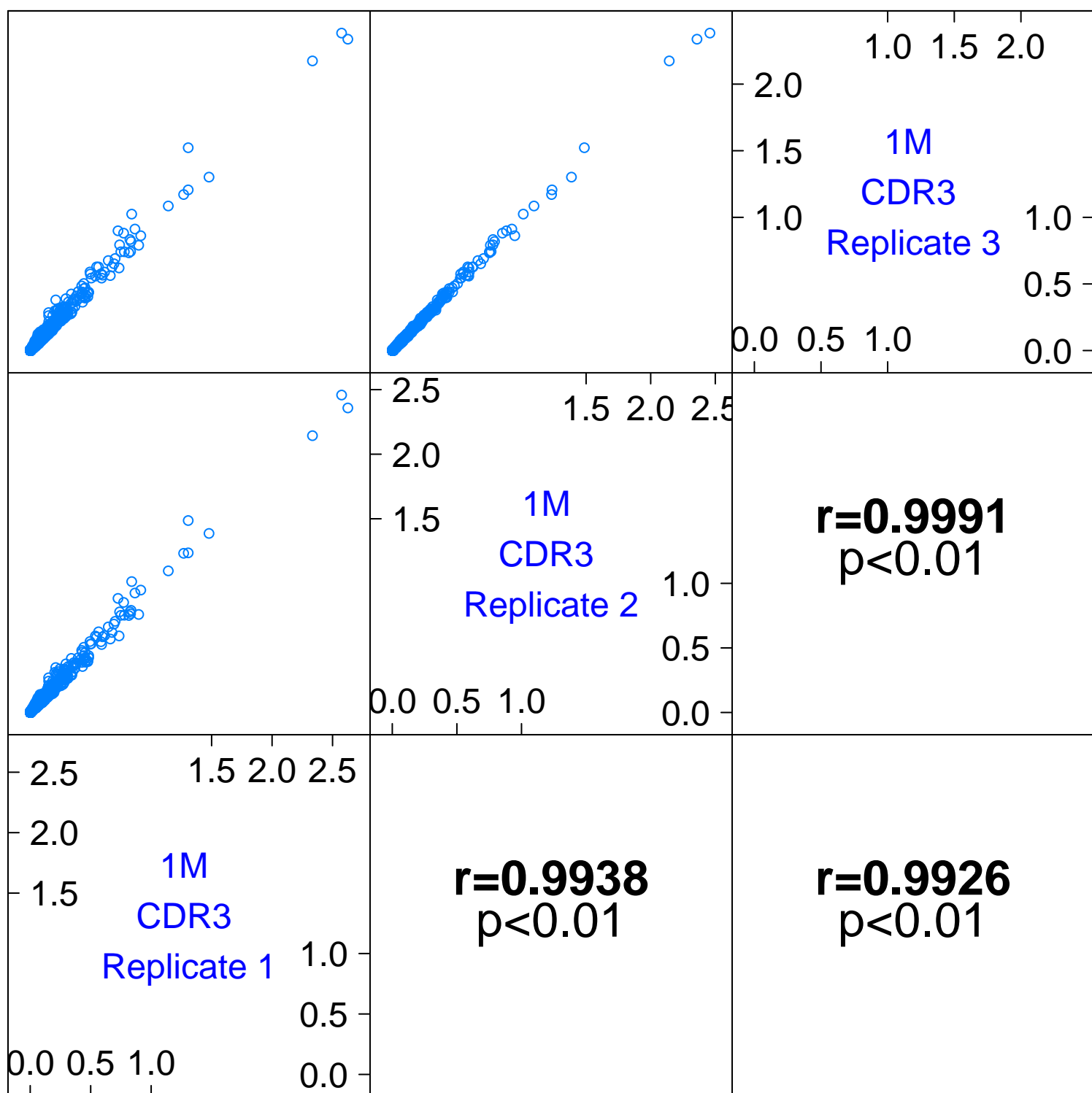

B

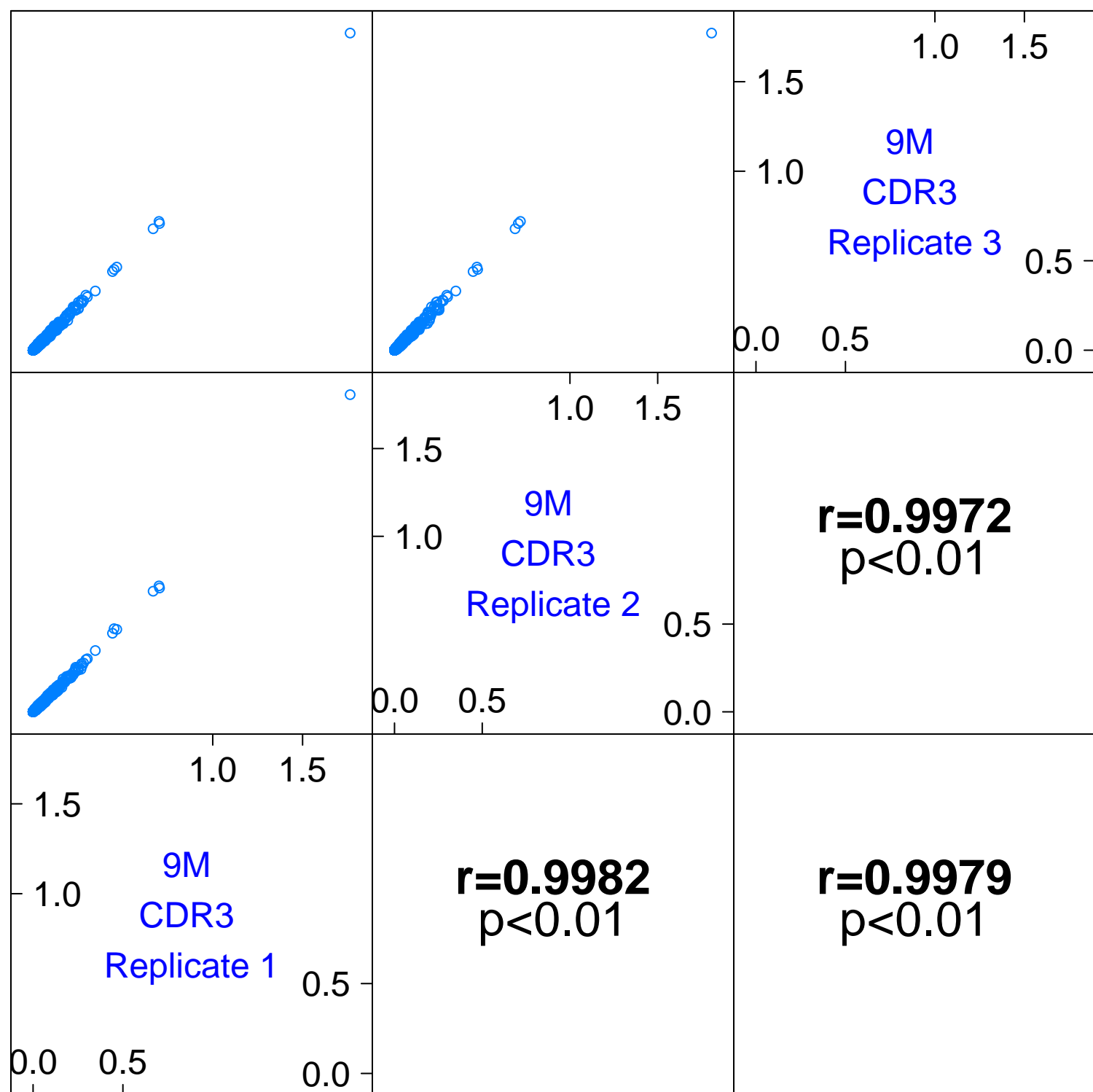

C

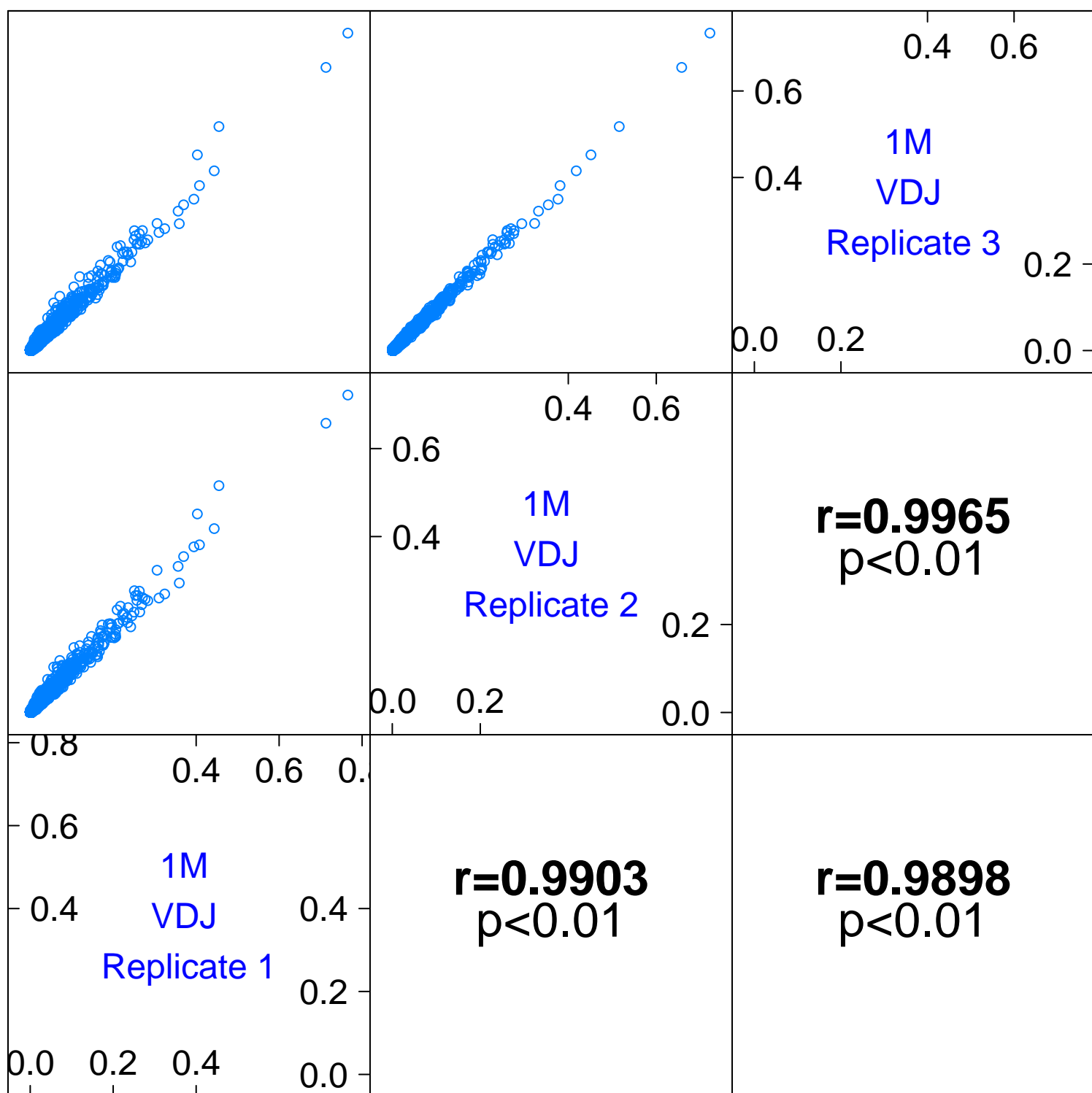

D

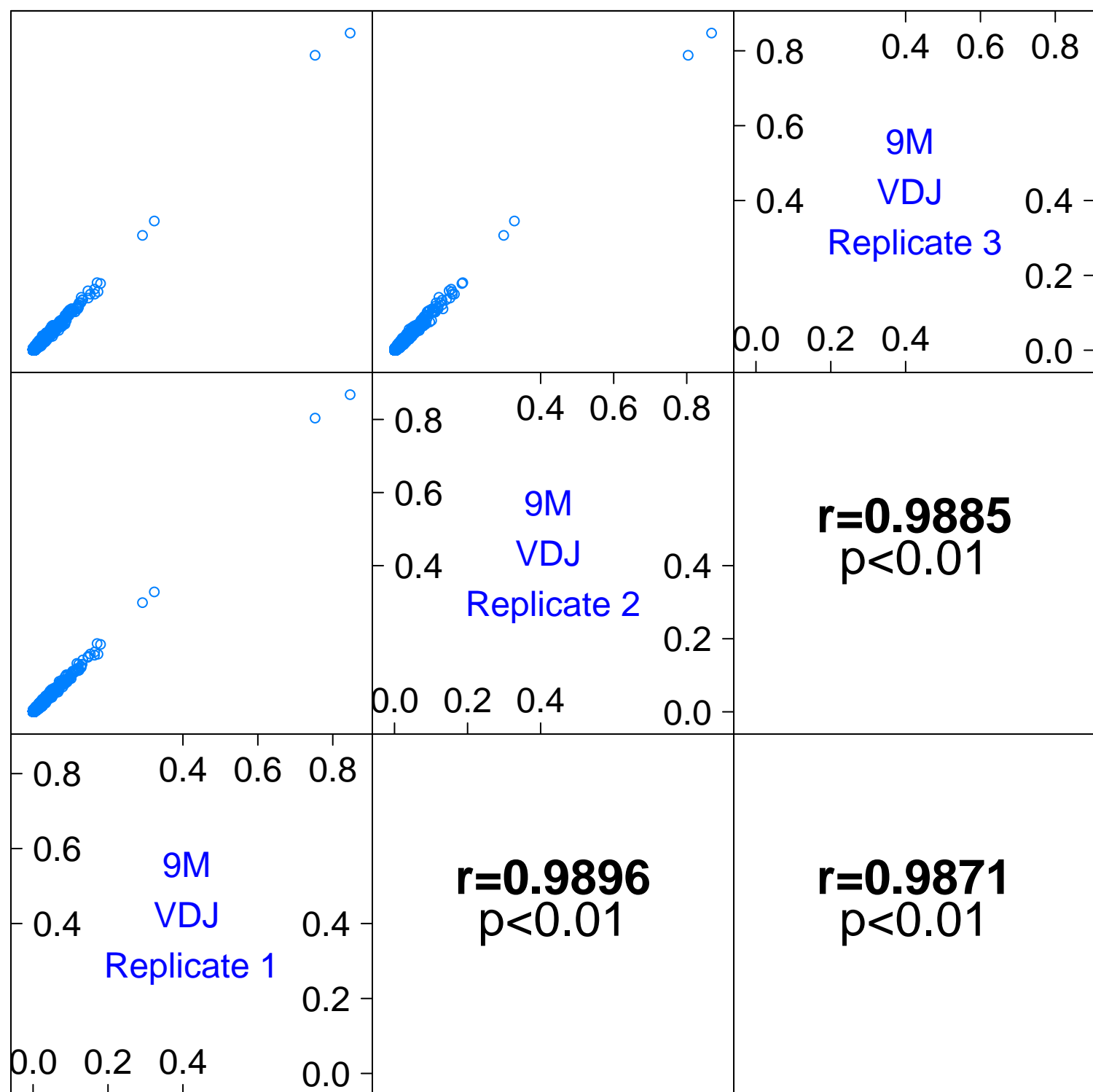

Supplement: Additional file 7: — Triplicate correlation of CDR3 and VDJ frequencies is high. CDR3 (A, B) and VDJ (C, D) frequencies were Pearson correlated (r) among triplicates (CDR3: r ≥ 0.9926, VDJ: r ≥ 0.9871) indicating the reproducibility of antibody repertoire sequencing. Only reliably detected CDR3 and VDJ sequences (Figure 3) were considered for the analysis shown. [file 12865_2014_40_MOESM7_ESM.pdf]

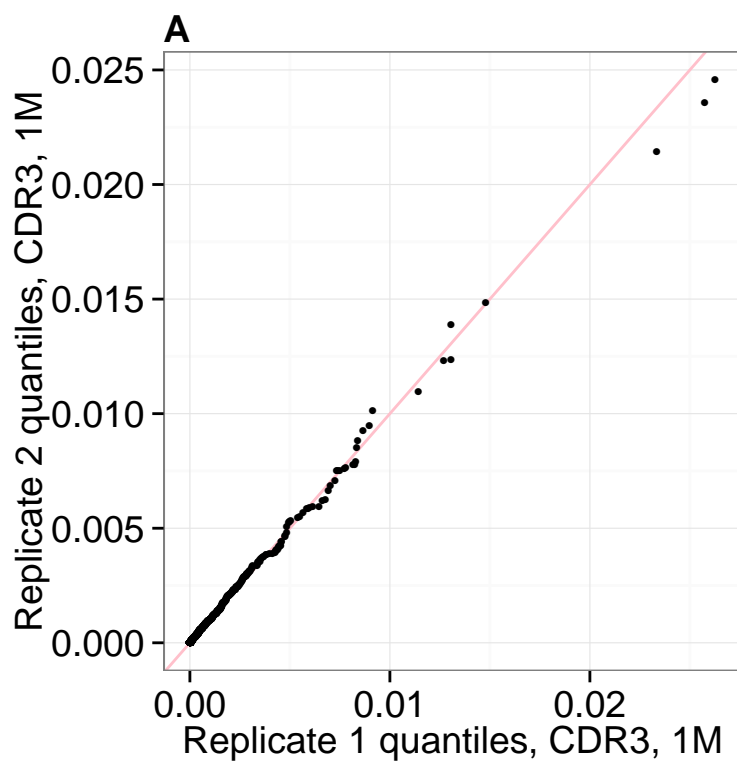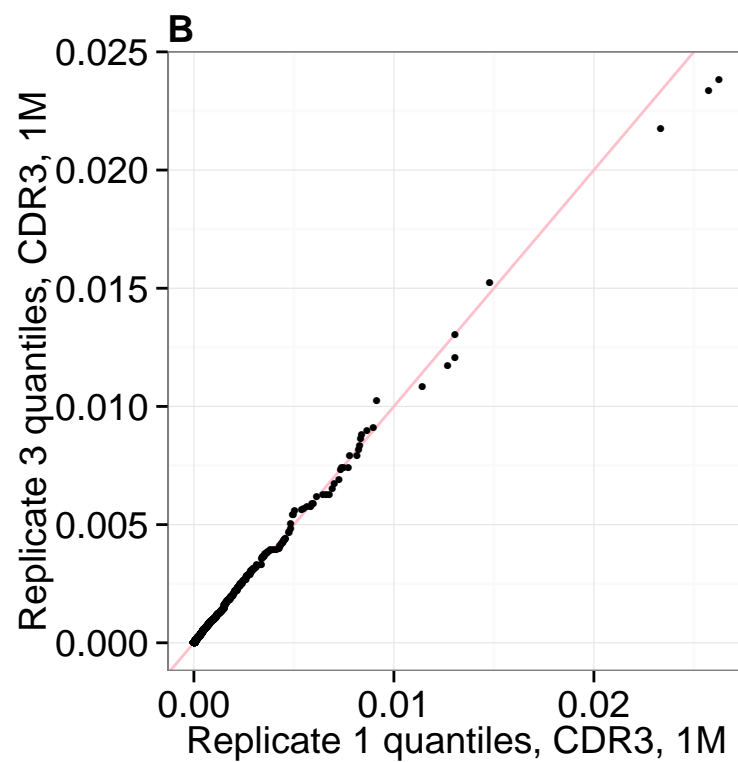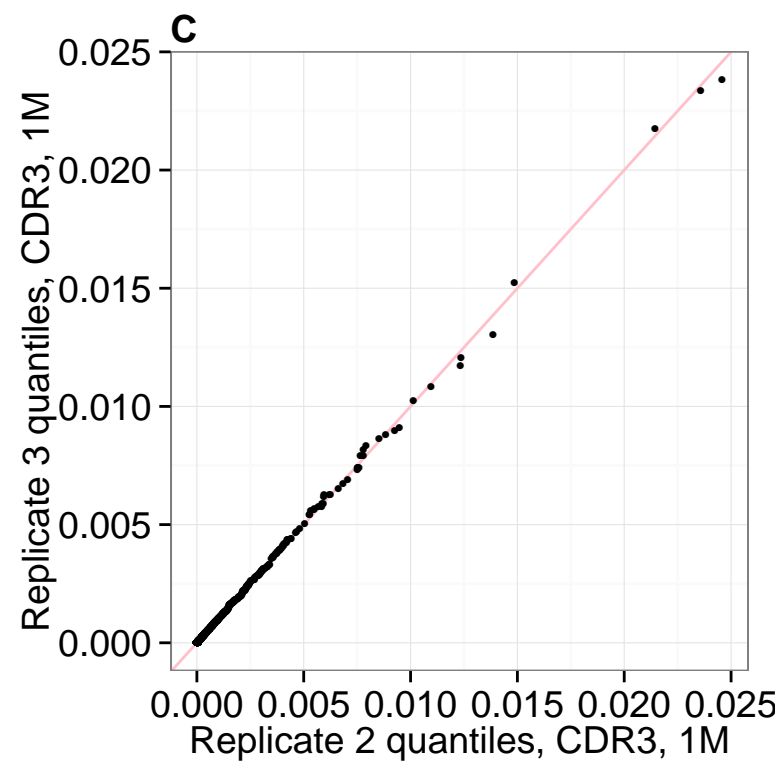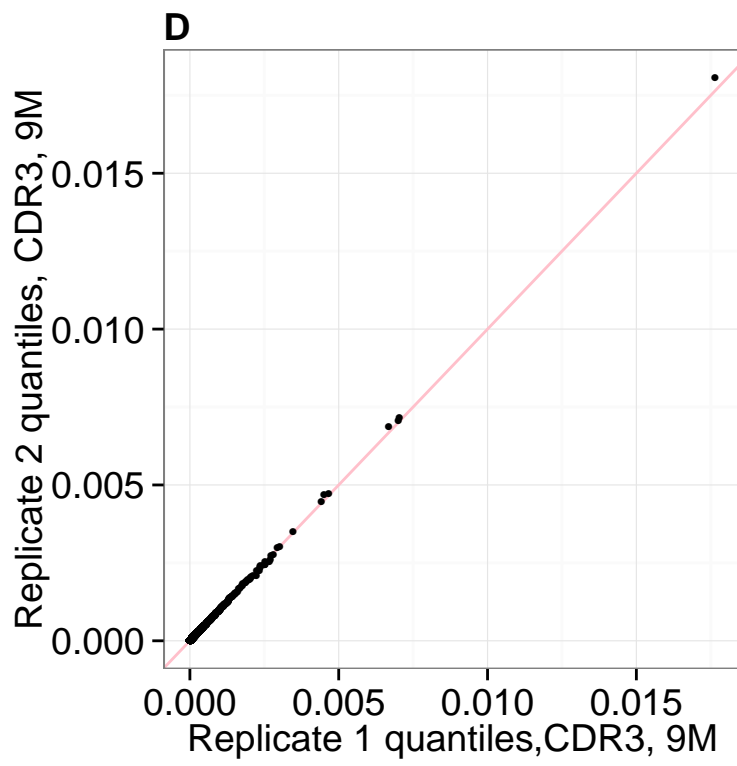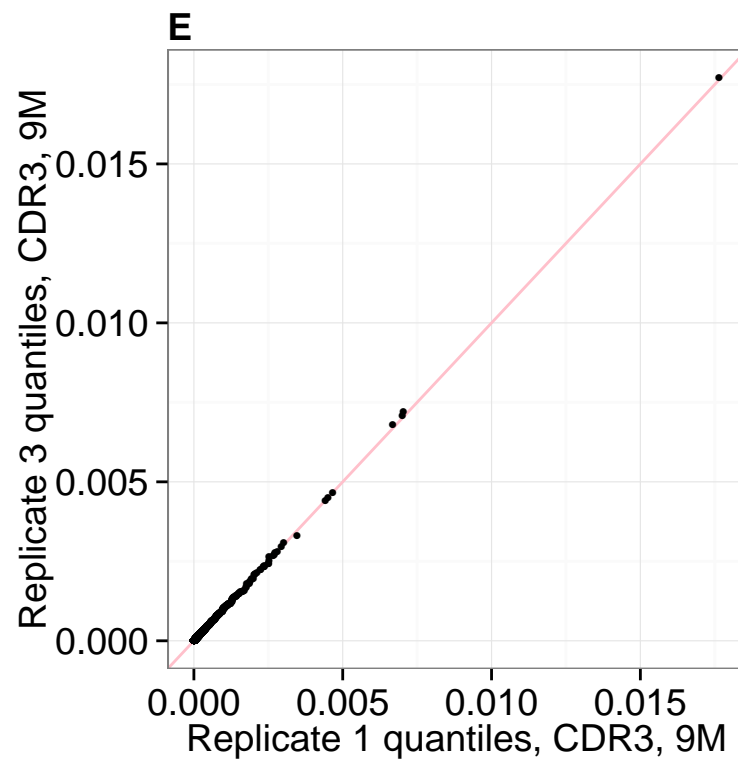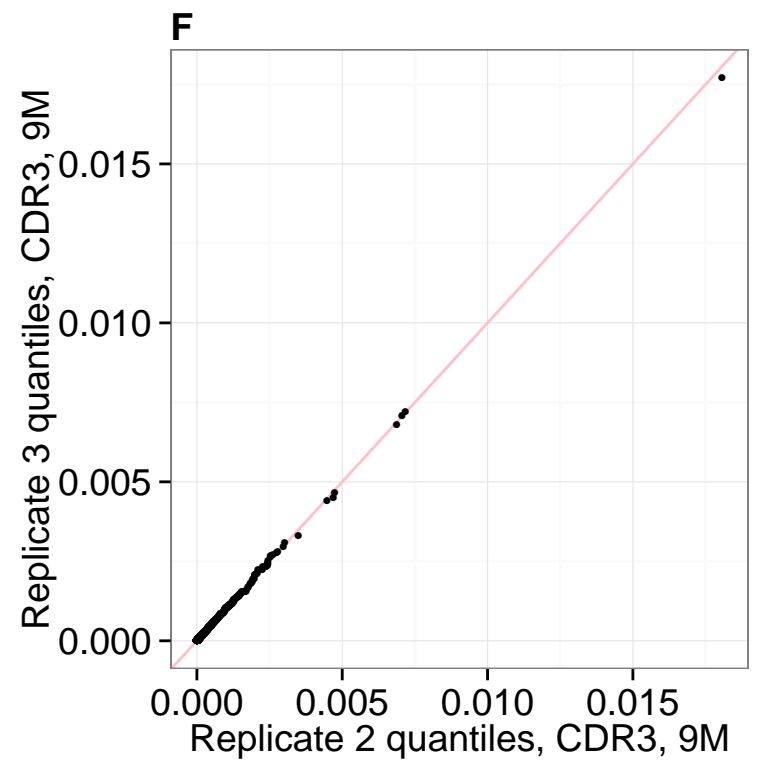

Supplement: Additional file 8: — Quantile-Quantile (Q-Q) plots of triplicates show that CDR3 frequency distributions (Additional file 5 ) are reproducible across diversity scenarios. Q-Q plots (1M: A, B, C; 9M: D, E, F) represent a graphical approach for comparing two frequency distributions by plotting their quantiles against each other. If the two distributions being compared are similar, the quantiles will lie on the indicated line. Only reliably detected CDR3 sequences (Figure 3) were considered for the analysis shown. [file 12865_2014_40_MOESM8_ESM.pdf]

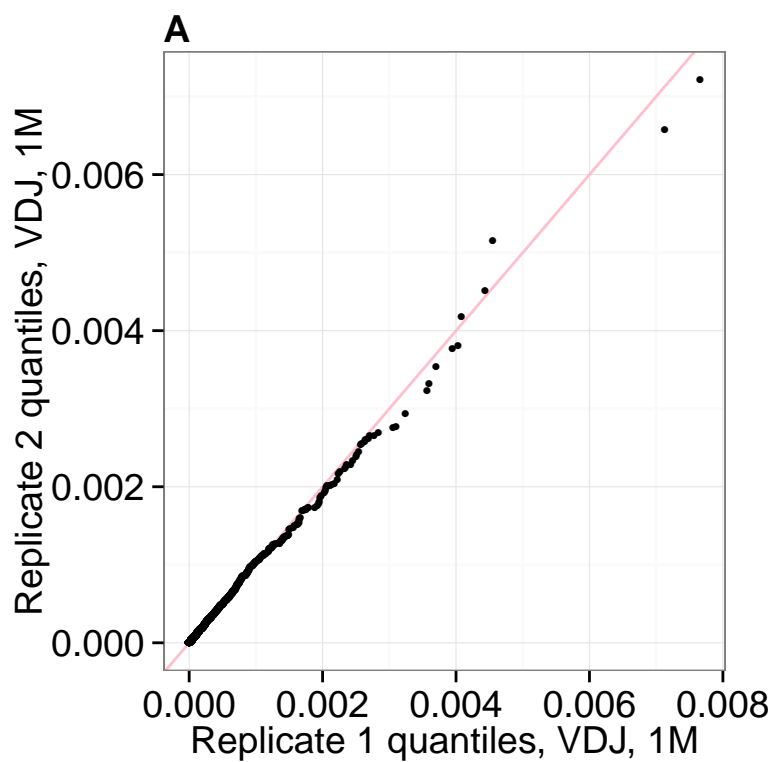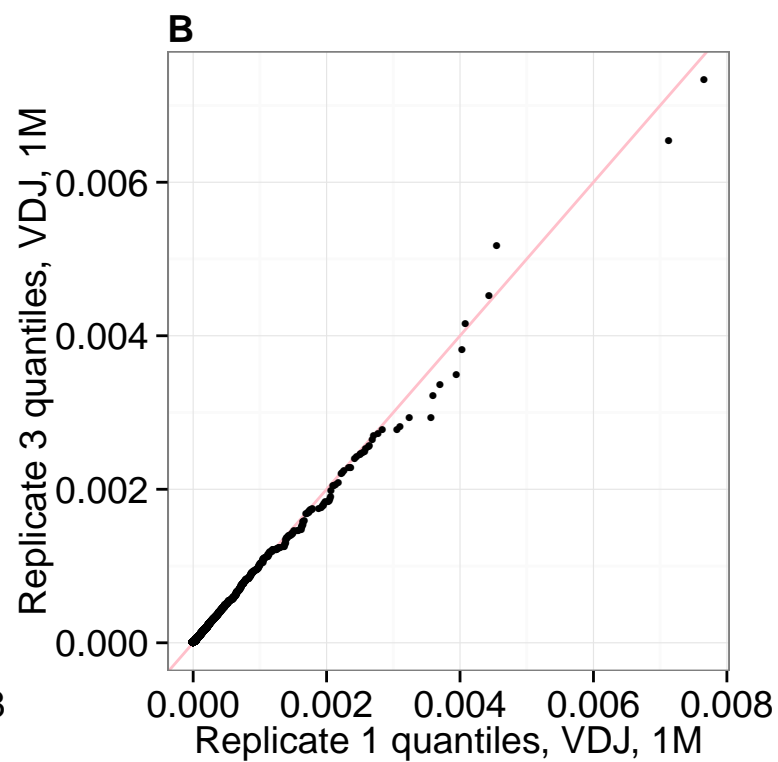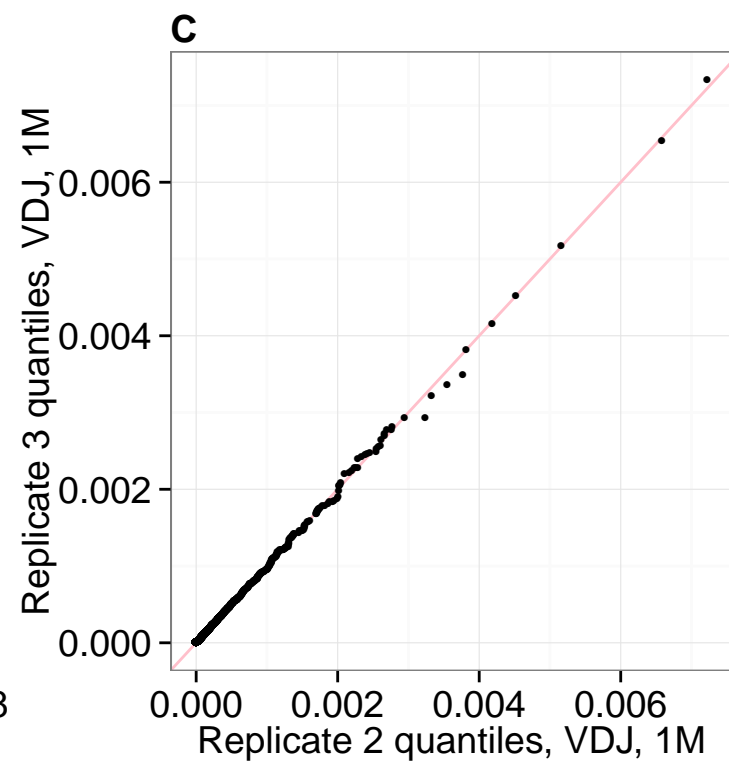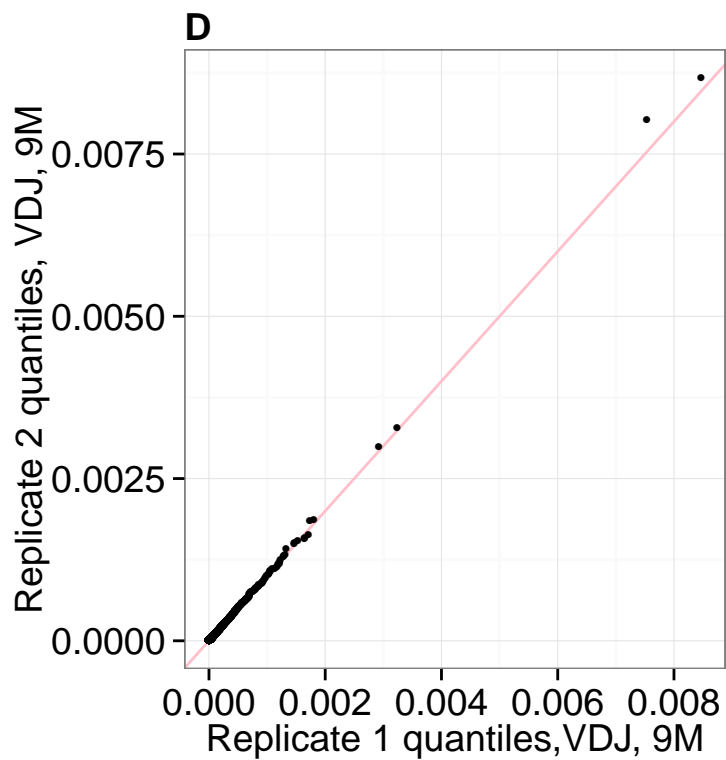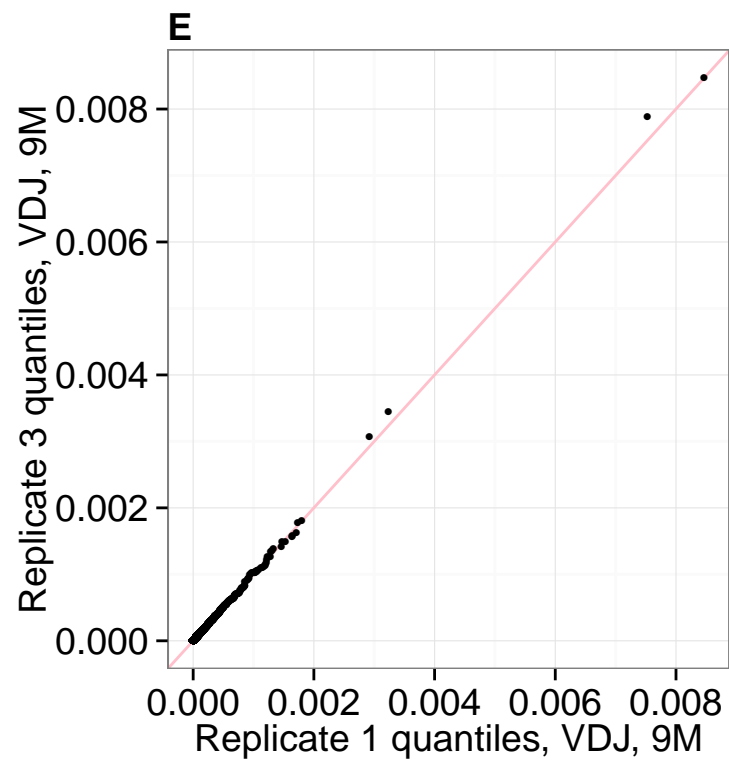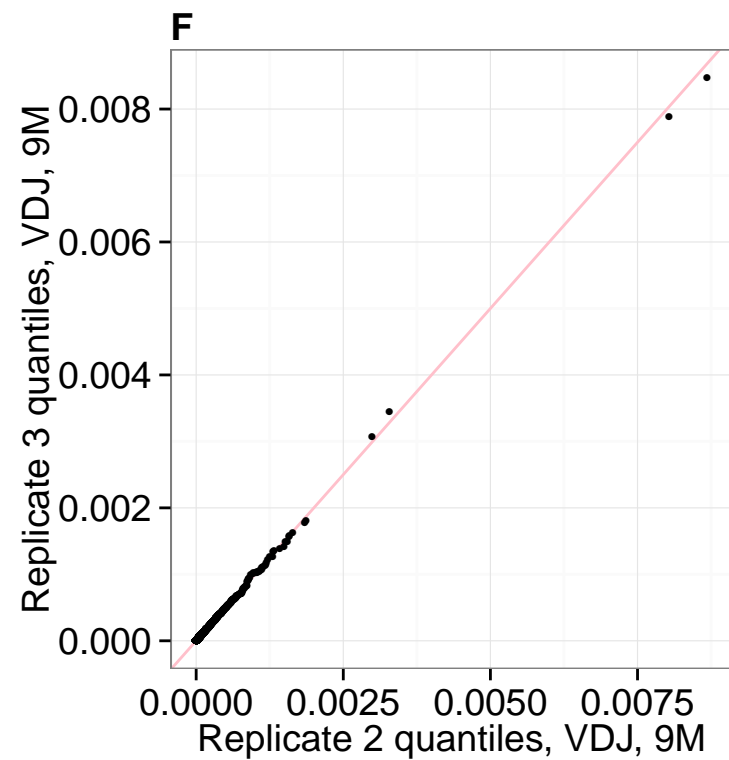

Supplement: Additional file 9: — Quantile-Quantile (Q-Q) plots of triplicates show that VDJ frequency distributions (Additional file 5 ) are reproducible across diversity scenarios. Only reliably detected VDJ sequences (Figure 3) were considered for the analysis shown (1M: A, B, C; 9M: D, E, F). [file 12865_2014_40_MOESM9_ESM.pdf]

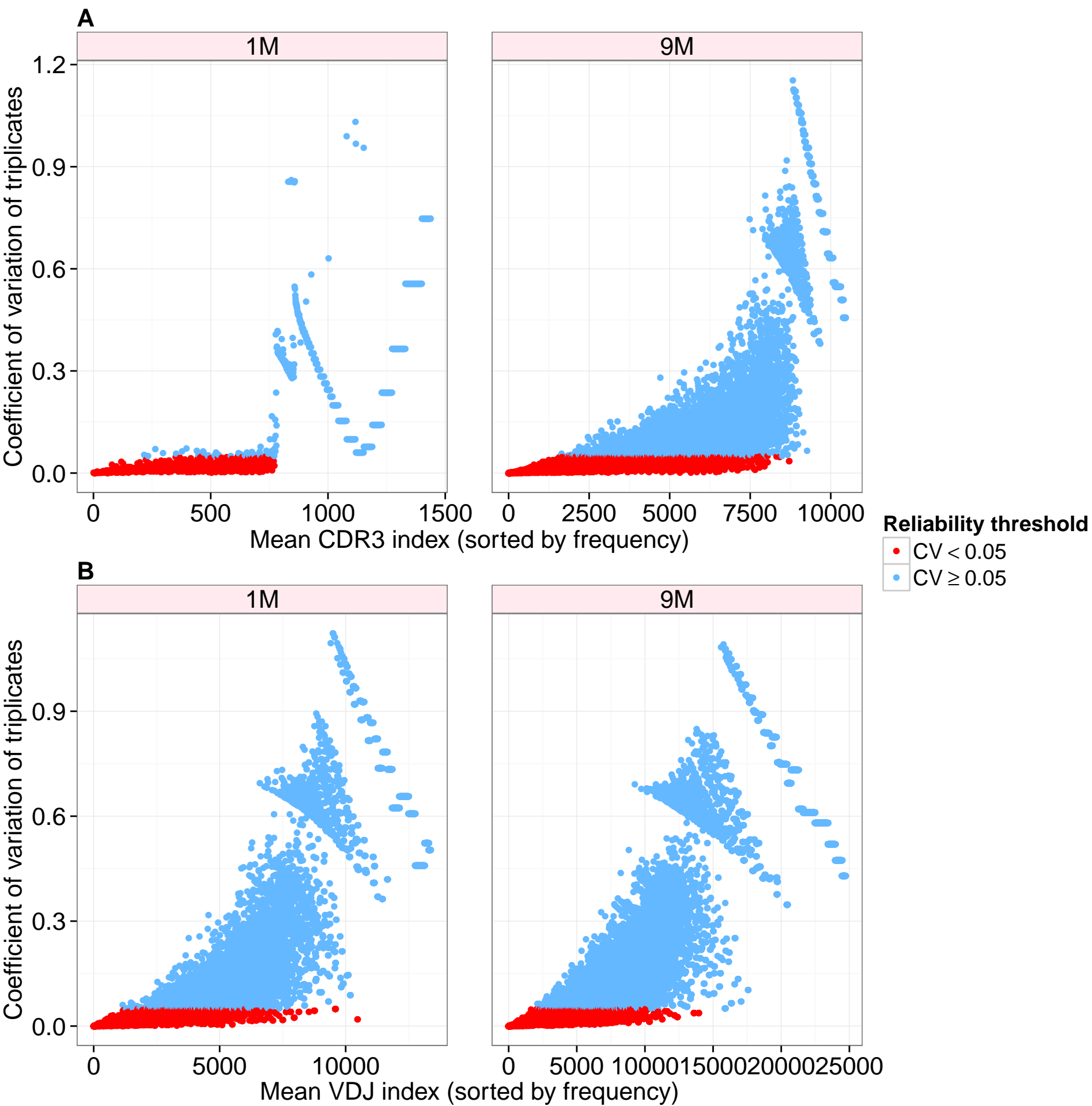

Supplement: Additional file 10: — Determination of reliability of CDR3 and VDJ ranking. (A, B) Taking advantage of triplicate sequencing, coefficients of variation (CV = SD/mean) based on ranks were determined for all reliably detected CDR3 or VDJ clones (as determined in Figure 3) and plotted in function of highest to lowest mean CDR3/VDJ frequency. CDR3/VDJ having a CV lower than 0.05 were regarded as reliably ranked and are shown in red. Absolute numbers of reliably ranked clones are: CDR3, 730/4,160 (1M/9M), VDJ, 3,708/5,169 (1M/9M). [file 12865_2014_40_MOESM10_ESM.pdf]

**A**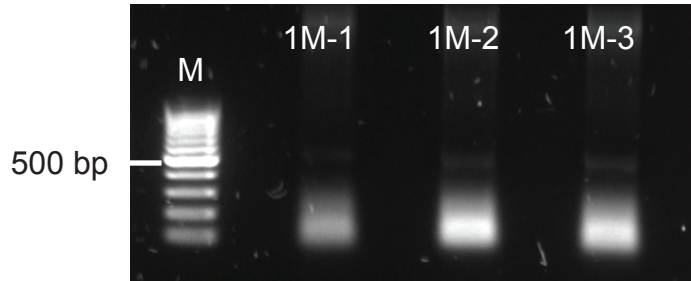**B**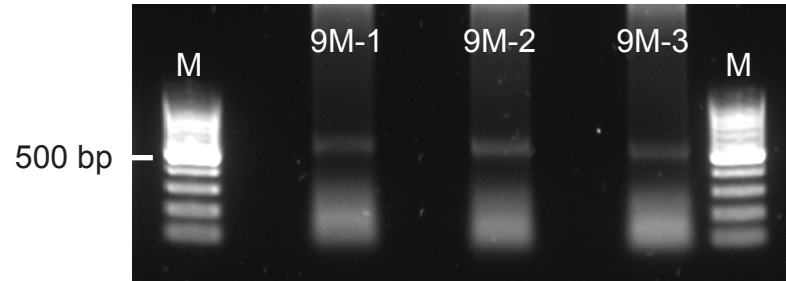

Supplement: Additional file 11: — Triplicate IgG amplicon libraries on 1% agarose gel for (A) 1M and (B) 9M diversity scenarios. Libraries were prepared each with cDNA equivalent to 500 ng of total pooled RNA of ASCs (see Methods). 570-bp sized amplicons were gel-extracted and purified. Legend: M – 100 bp DNA ladder. [file 12865_2014_40_MOESM11_ESM.pdf]

**A**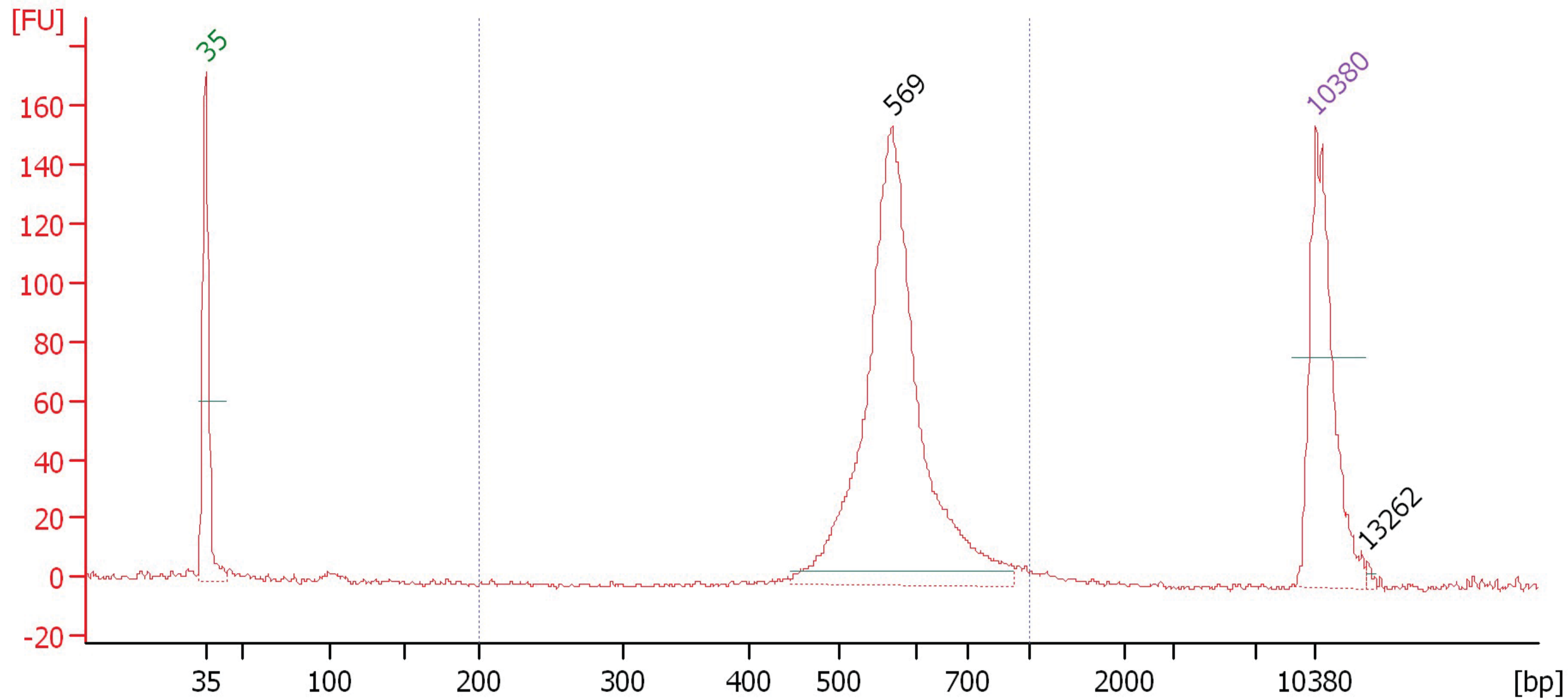**B**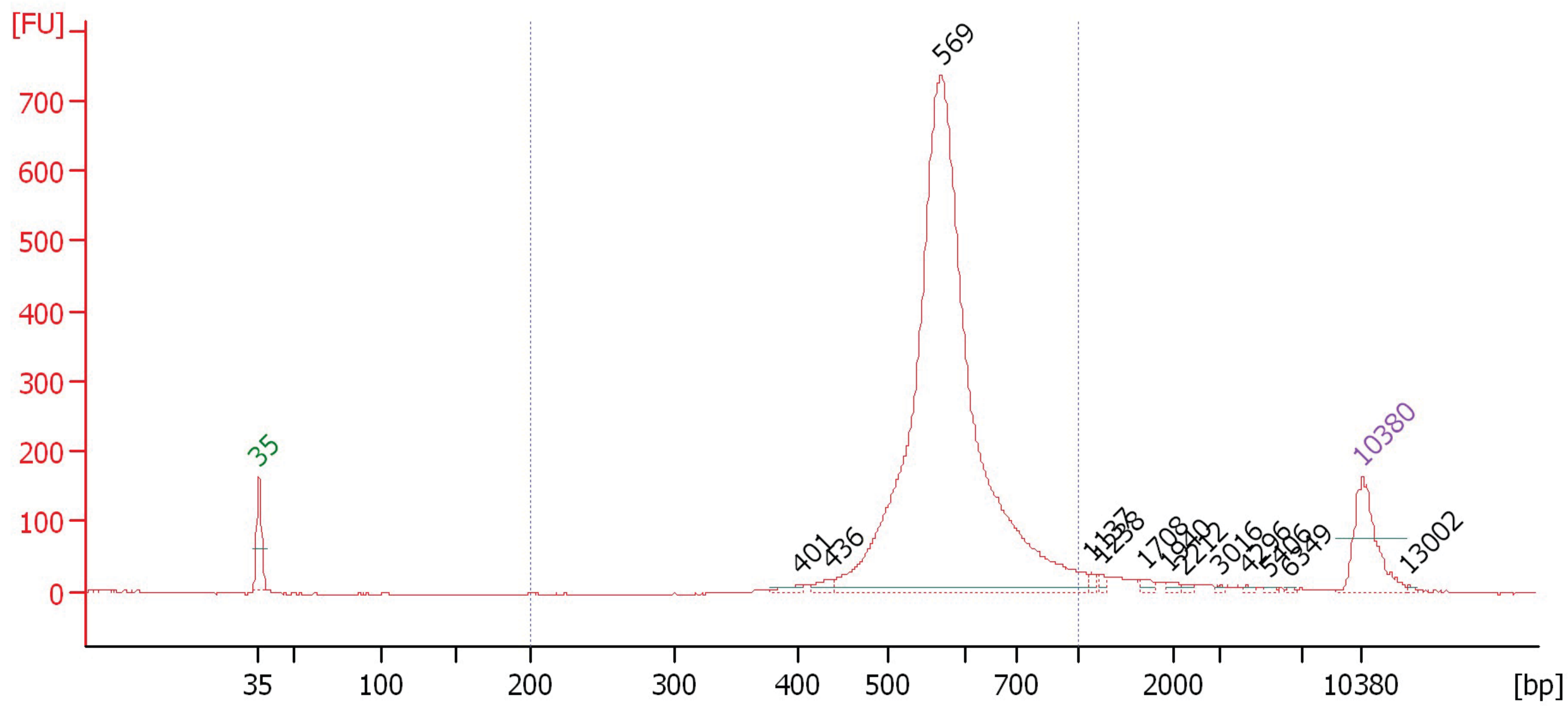

Supplement: Additional file 12: — Bioanalyzer electropherograms of (A) one 1M and (B) one 9M replicate after gel extraction. Single, clear peaks were detected for all triplicates of both diversity scenarios (other replicates not shown), resulting on average in 450/1,200 pg NGS-ready library for 1M/9M, respectively. [file 12865_2014_40_MOESM12_ESM.pdf]
