# Supplementary material for: Quantitative assessment of the robustness of next-generation sequencing of antibody variable gene repertoires from immunized mice
Source: BMC Immunol. 2014 Oct 16;15:40. doi: 10.1186/s12865-014-0040-5 (PMC4233042; doi:10.1186/s12865-014-0040-5)
Supplement: Additional file 3: — Read statistics. Quality Phred scores, returned 250 bp paired-end reads prior to PANDAseq pairing and IMGT annotation and detected CDR3s/VDJs prior to application of various cutoffs (≥2 and reliability cutoff established in Figure 3). For each cutoff, the total number of CDR3s/VDJs (“All”) as well as respective “unique” CDR3s/VDJs (species richness) are reported. Average numbers are reported as are the percentages of CDR3s/VDJs that passed cutoffs compared to the total number of CDR3s/VDJs. Percentages in brackets indicate (i) the ratio of PANDAseq-paired reads out of the returned 250 bp reads (column: “Returned reads”), (ii) or the ratio of CDR3s/VDJs out of the PANDAseq-paired reads (columns: “All CDR3s/VDJs”). Replicate 2 (1M) and 3 (9M) have been used in simulations shown in Figure 2. [file 12865_2014_40_MOESM3_ESM.docx]

| Replicates | Phred scores | Returned 250 bp paired-end reads  endreads | All CDR3s | All CDR3s:  unique | All CDR3s *≥* 2 | All CDR3s  *≥* 2: unique | All CDR3s *≥*  cut-off | All CDR3s  *≥* cut-off: unique | All VDJs | All VDJs:  unique | All VDJs *≥* 2 | All VDJs *≥* 2:  unique | All VDJs*≥* cut- off | All VDJs*≥* cut- off: unique |
| --- | --- | --- | --- | --- | --- | --- | --- | --- | --- | --- | --- | --- | --- | --- |
|  |  |  |  |  |  |  |  |  |  |  |  |  |  |  |
|  |  |  |  |  |  |  |  |  |  |  |  |  |  |  |
| 1M, Replicate 1 | 36.16 | 2,171,738 (99%) | 1,040,169 (97%) | 42,199 | 1,010,561 | 12,591 | 966,422 | 1,427 | 991,132 (92%) | 365,744 | 675,314 | 49,926 | 565,172 | 10,741 |
| 1M, Replicate 2 | 33.15 | 3,139,850 (85%) | 1,231,251 (93%) | 57,025 | 1,189,447 | 15,221 | 1,117,463 | 862 | 1,170,374 (88%) | 432,236 | 795,466 | 57,328 | 659,408 | 10,731 |
| 1M, Replicate 3 | 33.65 | 2,906,522 (86%) | 1,166,955 (93%) | 49,697 | 1,131,269 | 14,011 | 1,064,800 | 791 | 1,110,489 (88%) | 404,411 | 760,698 | 54,620 | 636,365 | 11,055 |
| 1M, Average of | 34.32 | 2,739,370 | 1,146,125 | 49,640 | 1,110,425 | 13,941 | 1,049,561 | 1,026 | 1,090,665 | 400,797 | 743,826 | 53,958 | 620,315 | 10,842 |
| replicates |  |  |  |  |  |  |  |  |  |  |  |  |  |  |
| 1M, Average of |  |  |  |  | 96.90 | 28.24 | 91.64 | 2.16 |  |  | 68.20 | 13.47 | 56.89 | 2.72 |
| replicates: per- |  |  |  |  |  |  |  |  |  |  |  |  |  |  |
| centages |  |  |  |  |  |  |  |  |  |  |  |  |  |  |
| 9M, Replicate 1 | 35.05 | 2,933,918 (93%) | 1,297,575 (95%) | 93,350 | 1,227,675 | 23,450 | 1,190,239 | 9,414 | 1,233,002 (90%) | 617,118 | 706,944 | 91,060 | 487,636 | 18,765 |
| 9M, Replicate 2 | 35.31 | 5,559,528 (96%) | 2,522,724 (95%) | 163,382 | 2,399,840 | 40,498 | 2,300,146 | 9,458 | 2,392,020 (90%) | 1,116,976 (90%) | 1,429,506 | 154,462 | 919,307 | 18,928 |
| 9M, Replicate 3 | 35.32 | 2,935,300 (95%) | 1,333,794 (96%) | 99,700 | 1,262,637 | 24,393 | 1,222,160 | 9,460 | 1,270,608 (91%) | 638,059 | 725,292 | 92,743 | 496,895 | 18,466 |
| 9M, Average of | 35.23 | 3,809,582 | 1,719,414 | 118,811 | 1,630,050 | 29,447 | 1,570,850 | 9,453 | 1,654,322 | 790,717 | 953,914 | 112,755 | 634,612 | 18,719 |
| replicates |  |  |  |  |  |  | , |  |  |  |  |  |  |  |
| 9M, Aver- |  |  |  |  | 94.70 | 24.79 | 91.42 | 8.46 |  |  | 58.06 | 14.37 | 39.03 | 2.54 |
| age of repli- |  |  |  |  |  |  |  |  |  |  |  |  |  |  |
| cates: percentages |  |  |  |  |  |  |  |  |  |  |  |  |  |  |
