# Supplementary material for: Quantitative assessment of the robustness of next-generation sequencing of antibody variable gene repertoires from immunized mice
Source: BMC Immunol. 2014 Oct 16;15:40. doi: 10.1186/s12865-014-0040-5 (PMC4233042; doi:10.1186/s12865-014-0040-5)
Supplement: Additional file 4: — CDR3 repertoires from 1M were more polarized than those from 9M and CDR3 repertoires were consistently more polarized than VDJ repertoires (1M, 9M). The Berger-Parker index was used to measure the polarization of repertoires [84] and is determined as the ratio N max/N , where N max is the abundance of the top CDR3 or VDJ clone, and N is the sum of abundances of all CDR3 or VDJ sequences in a replicate. The index was calculated both for frequency distributions containing either all CDR3 or VDJ clones with an abundance of 2 or higher and those CDR3 or VDJ clones passing the reliability cutoff established in Figure 3. [file 12865_2014_40_MOESM4_ESM.docx]

| Replicates | CDR3*≥*2 | VDJ*≥*2 | CDR3*≥*cut-off | VDJ*≥*cut-off |
| --- | --- | --- | --- | --- |
| 1M, Replicate 1 | 0.0251 | 0.0064 | 0.0263 | 0.0077 |
| 1M, Replicate 2 | 0.0231 | 0.0060 | 0.0246 | 0.0072 |
| 1M, Replicate 3 | 0.0224 | 0.0061 | 0.0238 | 0.0073 |
| 9M, Replicate 1 | 0.0171 | 0.0058 | 0.0176 | 0.0085 |
| 9M, Replicate 2 | 0.0173 | 0.0056 | 0.0181 | 0.0087 |
| 9M, Replicate 3 | 0.0172 | 0.0058 | 0.0177 | 0.0085 |
